# Supplementary figures and images for: Multi-study Integration of Brain Cancer Transcriptomes Reveals Organ-Level Molecular Signatures
Source: PLoS Comput Biol. 2013 Jul 25;9(7):e1003148. doi: 10.1371/journal.pcbi.1003148 (PMC3723500; doi:10.1371/journal.pcbi.1003148)

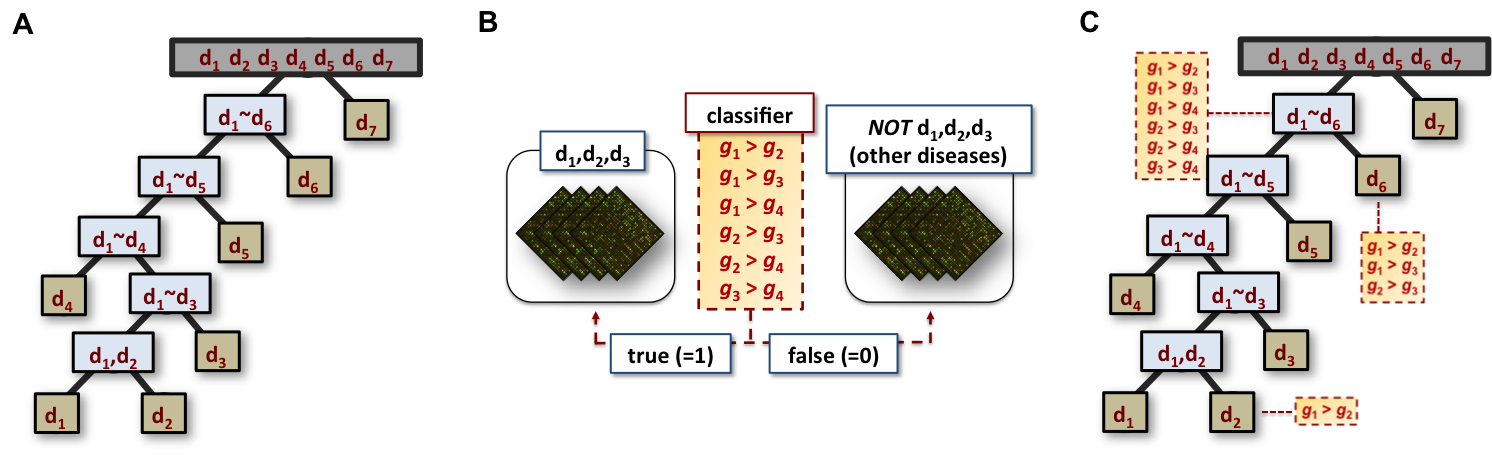

Supplement: Figure S1 — The overall method of ISSAC can be summarized into three main steps. A ISSAC constructs the framework for brain cancer diagnosis – a tree-structured hierarchy of all brain cancer phenotypes built using an agglomerative hierarchical clustering algorithm on gene expression training data. B Training on gene-expression data from all brain phenotypes, ISSAC identifies disjoint, gene-pair classifiers at all nodes (excluding the root) and edges of the diagnostic hierarchy, and accumulates them into their respective marker panels. The chosen pairs are the ones that best differentiate between the phenotype sets, and are based entirely on the reversal of relative expression. C ISSAC uses the gene-pair classifiers for class prediction. Briefly, given a gene expression profile, ISSAC executes the node classifiers in a hierarchical, top-down fashion within the disease diagnostic hierarchy to identify the phenotype(s) whose class-specific signature(s) is present. In case of multiple class candidates (i.e. node classifiers for multiple leaves are positive), the ambiguity is resolved by aggregating all the decision-tree classifiers into a classification decision-tree, thereby leading any expression signature down one unique path toward a single phenotype. (TIFF) [file pcbi.1003148.s001.tiff]

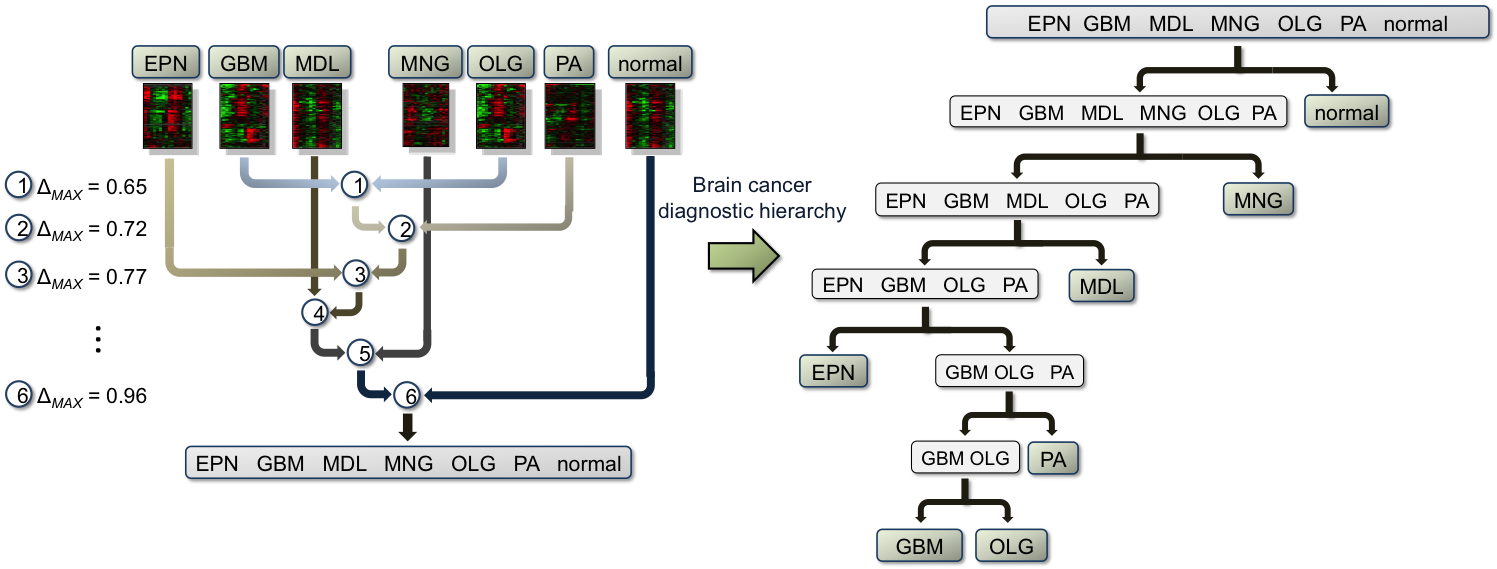

Supplement: Figure S2 — Brain phenotypes are grouped into a global diagnostic hierarchy, which allows an intuitive representation of the classification process. The diagnostic hierarchy is built using a data-driven, iterative approach, and is free of manual, ad-hoc construction. In each iteration, two classes, or two groups of classes, with the lowest TSP score (Materials and Methods and Text S1) among all pair-wise comparisons, come together to form a node. This approach optimizes overall classification by placing the more challenging decisions further away from the base of the tree (i.e. root), thereby ensuring only the minimum misclassifications percolate down the tree. The final form of the brain phenotype diagnostic hierarchy represents a hierarchical structure of nested partitions, where the multi-class problem is decomposed into smaller and smaller groups using a sequence of diagnostic decision rules. (TIFF) [file pcbi.1003148.s002.tiff]

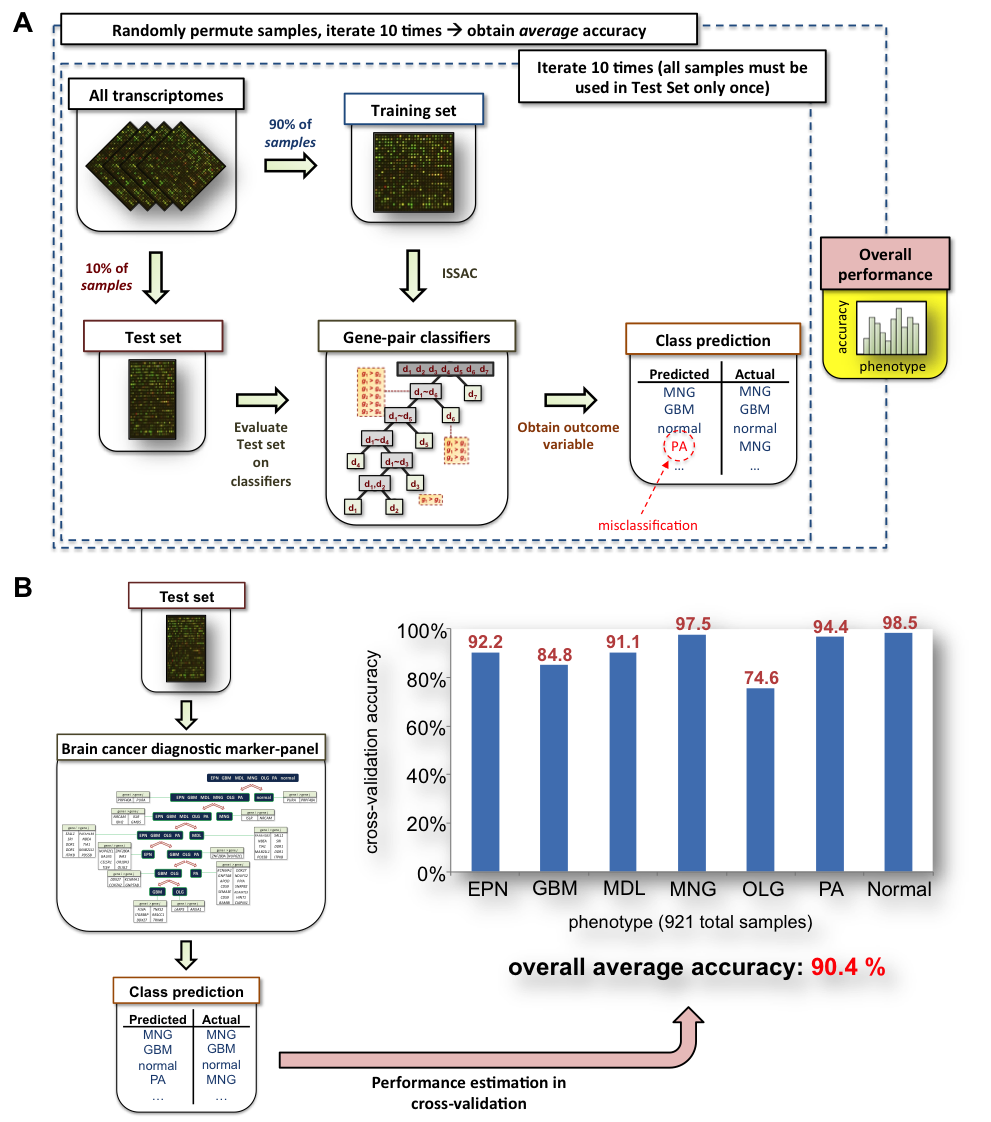

Supplement: Figure S3 — Performance evaluation using ten-fold cross-validation. A Ten-fold cross-validation is conducted ten times to obtain the average accuracy. In every iteration of cross-validation, the order of samples within a particular class are randomly permuted before training/test set allocations. B Our marker panel achieved a 90.4% average of phenotype-specific classification accuracies, showing strong promise against a multi-category, multi-dataset background at the gene expression level. (TIFF) [file pcbi.1003148.s003.tiff]

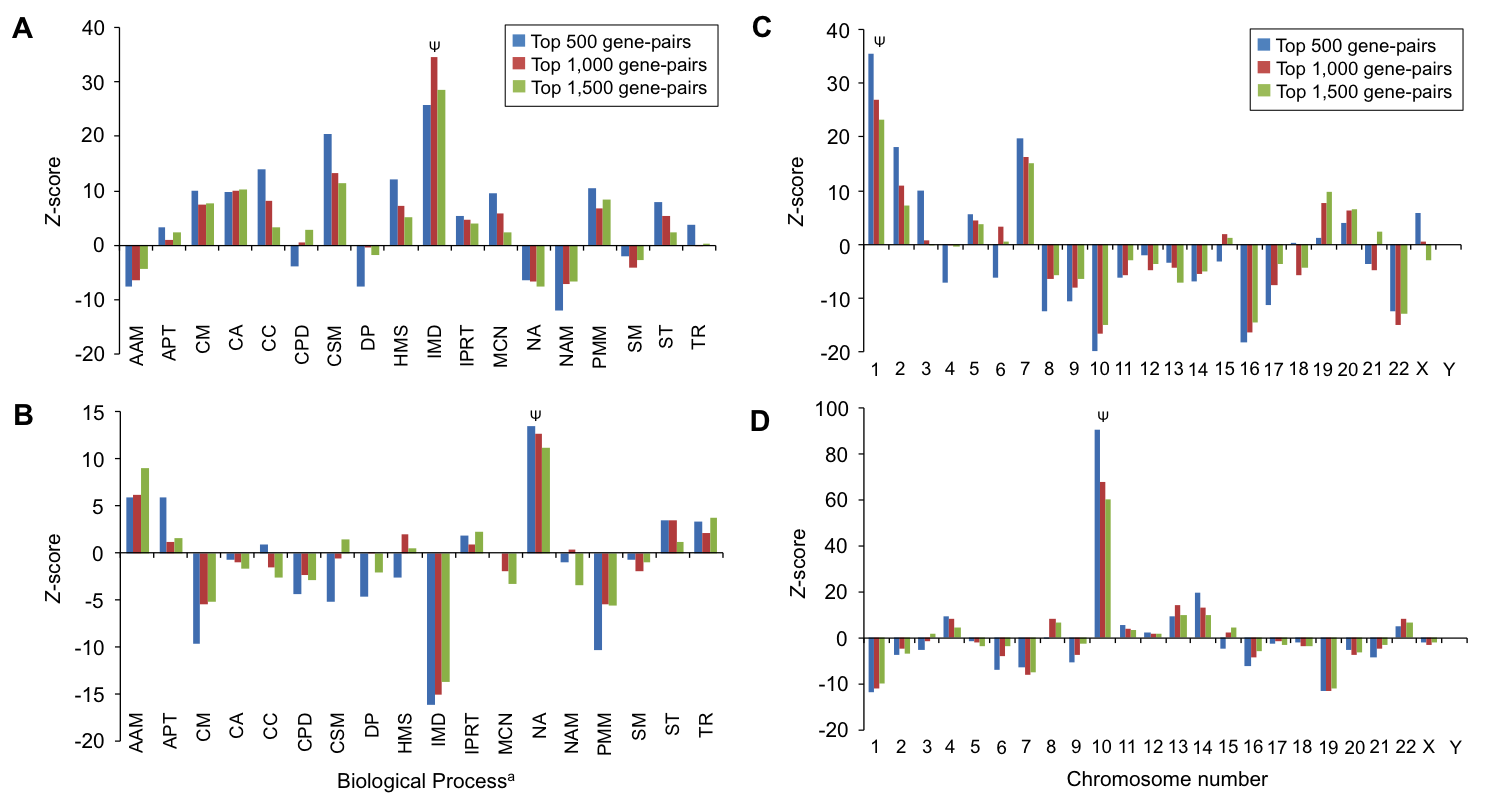

Supplement: Figure S4 — Statistical enrichment analysis on PANTHER database biological processes and chromosome numbers of the top 500, 1,000, and 1,500 gene-pair classifiers for GBM vs. OLG. A ‘Immunity and Defense’ was the most enriched biological process for ‘gene-set i’, reflecting the chronic inflammatory conditions inside the GBM tumor. B ‘Neuronal Activities’ was the most enriched biological process for ‘gene-set j’, reflecting decrease in neuronal behavior and possibly other brain cell activity inside the GBM tumor. The genes in ‘gene-set i’ and ‘gene-set j’ were the most enriched in C Chromosome 1 and D Chromosome 10, respectively, reflecting the major chromosome aberrations of the two brain cancers. Ψ delineates the most enriched category. aBiological process abbreviation (name): AAM (Amino acid metabolism), APT (Apoptosis), CM (Carbohydrate metabolism), CA (Cell adhesion), CC (Cell cycle), CPD (Cell proliferation and differentiation), CSM (Cell structure and motility), DP (Developmental processes), HMS (Homeostatis), IMD (Immunity and defense), IPRT (Intracellular protein transport), MCN (Muscle contraction), NA (Neuronal activities), NAM (Nucleic acid metabolism), PMM (Protein metabolism and modification), SM (Sulfur metabolism), ST (Signal transduction), and TR (Transport). (TIFF) [file pcbi.1003148.s004.tiff]

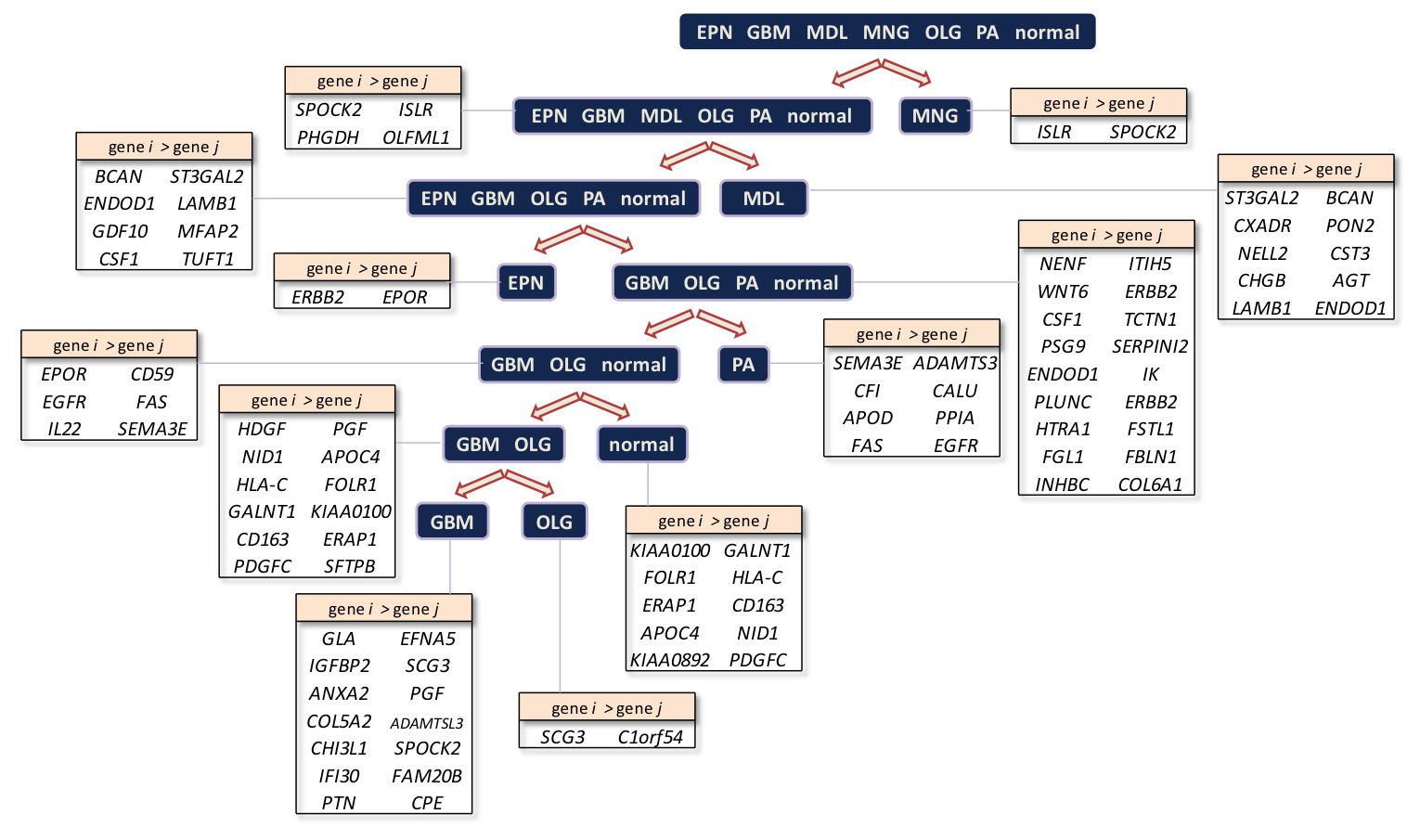

Supplement: Figure S5 — Gene-pair classifiers based on only the genes that encode extracellular products. Gene pairs are shown at their corresponding nodes in the brain disease diagnostic hierarchy. The corresponding node-based marker panel consists of 41 classifier pairs and 71 unique classifier features. (TIFF) [file pcbi.1003148.s005.tiff]
