# Supplementary material for: Multi-study Integration of Brain Cancer Transcriptomes Reveals Organ-Level Molecular Signatures
Source: PLoS Comput Biol. 2013 Jul 25;9(7):e1003148. doi: 10.1371/journal.pcbi.1003148 (PMC3723500; doi:10.1371/journal.pcbi.1003148)
Supplement: Table S1 — Phenotype specimen descriptions and main results for all GEO accessions used in this study. (PDF) [file pcbi.1003148.s006.pdf]

Table S1. Phenotype specimen descriptions and main results for all GEO accessions used in this study.

Phenotype Name	GEO accession #	First Author (publication year)	Phenotype specimen description	Main results
Ependymoma	GSE16155	Donson (2009)	• Human ependymoma tumor resections	• Genes associated with nonrecurrent ependymoma were predominantly immune function-related<br>• Histological analysis of a subset of immune function genes revealed that their expression was restricted to tumor-infiltrating subpopulation<br>• Up-regulation of immune function genes is the predominant ontology associated with a good prognosis in ependymoma
	GSE21687	Johnson (2010)	• Human ependymomas comprised of minimum 85% tumour cells	• Identified subgroups of ependymoma, and subgroup-specific gene amplifications and deletions<br>• Comparative transcriptomics between human tumors and mouse neural stem cells generated mouse models of ependymoma with matching molecular expression patterns<br>• Developed a novel cross-species genomic approach to match subgroup-specific driver mutations with cellular compartments to model cancer subgroups
Glioblastoma Multiforme	GSE4412	Freije (2004)	• Diffuse infiltrating gliomas	• Gene expression-based grouping of tumors is a more powerful survival predictor than histologic grade or age<br>• The expression patterns of 44 genes classify gliomas into previously unrecognized biological and prognostic groups<br>• Large-scale gene expression analysis and subset analysis of gliomas reveals unrecognized heterogeneity of tumors
	GSE4271	Phillips (2006)	• Primary high-grade gliomas and matched recurrences	• Novel prognostic subclasses of high-grade astrocytoma closely resemble stages in neurogenesis<br>• One tumor class displaying neuronal lineage markers shows longer survival, while two tumor classes enriched for neural stem cell markers display equally short survival<br>• Poor prognosis subclasses exhibit either markers of proliferation or of angiogenesis and mesenchyme<br>• A robust two-gene prognostic model utilizing PTEN and DLL3 expression suggests that Akt and Notch signaling are hallmarks of poor prognosis versus better prognosis gliomas, respectively
	GSE8692	Liu (2007)	• Primary low/high grade gliomas	• Measured genome-wide mRNA expression levels and miRNA profiles by microarray analysis and RT-PCR, respectively<br>• Correlation coefficients were determined for all possible mRNA-miRNA pairs<br>• A subset of high correlated pairs were experimentally validated by overexpressing or suppressing a miRNA and measuring the correlated mRNAs
	GSE9171	Wiedemeyer (2008)	• Glioblastoma tumors	• A nonheuristic genome topography scan (GTS) algorithm was developed to characterize the patterns of genomic alterations in human glioblastoma (GBM)<br>• A codeletion pattern found among closely related INK genes in the GBM oncogenome challenges the prevailing single-hit model of RB pathway inactivation<br>• Results suggest a feedback regulatory circuit in the astrocytic lineage and demonstrate a bona fide tumor suppressor role for *p18*^{INK4C} in human GBM
	GSE4290	Sun (2006)	• Primary gliomas and nontumor brain samples	• Stem cell factor (SCF) activates brain microvascular endothelial cells in vitro and induces a potent angiogenic response in vivo<br>• SCF downregulation inhibits tumor-mediated angiogenesis and glioma growth, whereas SCF overexpression is associated with shorter survival in malignant glioma patients<br>• The SCF/c-Kit pathway plays an important role in tumor- and normal host cell-induced angiogenesis within the brain<br>• Anti-angiogenic strategies have great potential as a treatment approach for gliomas
Medulloblastoma	GSE10327	Kool (2008)	• Primary medulloblastomas and local relapses	• mRNA expression profiling and genomic hybridization arrays show 5 different types of medulloblastoma, each with characteristic pathway activation signatures and associated specific genetic defects<br>• Clinicopathological features significantly different between the 5 subtypes include metastatic disease, age at diagnosis, and histology
	GSE12992	Fattet (2009)	• Paediatric medulloblastomas	• Immunostaining of β-catenin showed extensive nuclear staining in a subset of samples<br>• Expression profiles show strong activation of the Wnt/β-catenin pathway, and complete loss of chromosome 6<br>• Patients with extensive nuclear staining were significantly older at diagnosis and were in complete remission after a mean follow-up of 75.7 months (range 27.5–121.2 months) from diagnosis<br>• Results confirm previous observations that CTNNB1-mutated tumours represent a distinct molecular subgroup of medulloblastomas with favourable outcome

Table S1. (Continued) Phenotype specimen descriptions and main results for all GEO accessions used in this study.

Phenotype Name	GEO accession #	First Author (publication year)	Phenotype specimen description	Main results
Meningioma	GSE4780	Scheck (2006)	• Benign (grade 1) and aggressive (grades 2 and 3) meningiomas	• The results of this study have not been publicly disclosed (only data are available)
	GSE9438	Claus (2008)	• Meningioma specimens without neurofibromatosis type 2, nonrecurrent	• Progesterone and estrogen hormone receptors (PR and ER, respectively) were measured via immunohistochemistry and compared with gene expression profiling results<br>• Gene expression seemed more strongly associated with PR status (+/-) than with ER status<br>• Genes in collagen and extracellular matrix pathways were most differentially expressed by PR status<br>• PR status may be a clinical marker for genetic subgroups of meningioma
	GSE 16581	Lee (2010)	• 85 meningioma samples of various grades	• Investigators performed retrospective global genetic and genomic analysis of 85 meningioma samples of various grades<br>• In addition to chromosome 22q loss, which was detected in the majority of clinical samples, chromosome 6q and 14q loss was significantly more common in recurrent tumors and was associated with anaplastic histology<br>• Five "classes" of meningiomas were detected by gene expression analysis that correlated with copy number alterations, recurrent status, and malignant histology<br>• Data from this study provide broad genomic information to further stratify meningioma patients into prognostic risk groups
Oligodendroglioma	GSE4412	Freije (2004)	• Primary high-grade gliomas and matched recurrences	• Novel prognostic subclasses of high-grade astrocytoma are identified and discovered to resemble stages in neurogenesis<br>• One tumor class displaying neuronal lineage markers shows longer survival, while two tumor classes enriched for neural stem cell markers display equally short survival<br>• Poor prognosis subclasses exhibit either markers of proliferation or of angiogenesis and mesenchyme<br>• A robust two-gene prognostic model utilizing PTEN and DLL3 expression suggests that Akt and Notch signaling are hallmarks of poor prognosis versus better prognosis gliomas, respectively
	GSE4290	Sun (2006)	• Primary gliomas and nontumor brain samples	• Stem cell factor (SCF) activates brain microvascular endothelial cells in vitro and induces a potent angiogenic response in vivo<br>• Downregulation of SCF inhibits tumor-mediated angiogenesis and glioma growth in vivo, whereas overexpression of SCF is associated with shorter survival in patients with malignant gliomas<br>• The SCF/c-Kit pathway plays an important role in tumor- and normal host cell-induced angiogenesis within the brain<br>• Antiangiogenic strategies have great potential as a treatment approach for gliomas
Pilocytic Astrocytoma	GSE 12907	Wong (2005)	• Juvenile pilocytic astrocytomas (JPAs)	• Genes involved in certain biological processes, including neurogenesis, cell adhesion, and central nervous system development, were significantly deregulated in JPA compared to those in normal cerebella<br>• Two major subgroups of JPA based on unsupervised hierarchical clustering<br>• JPA without myelin basic protein-positively stained tumor cells may have a higher tendency to progress
	GSE 5675	Sharma (2007)	• Primary pilocytic astrocytomas (PAs) arising sporadically and in patients with neurofibromatosis type 1 (NF1)	• No expression signature to discriminate clinically aggressive/recurrent tumors from indolent<br>• Unique gene expression pattern for PAs arising in patients with NF1<br>• Gene expression signature stratified PAs by location (supratentorial versus infratentorial)<br>• Glial tumors may share an intrinsic, lineage-specific molecular signature that reflects the brain region in which their nonmalignant predecessors originated
Normal Brain	GSE3526	Roth (2006)	• 20 anatomically distinct sites of the central nervous system (CNS)<br>• 8 autopsies for each CNS region<br>• Patient death was due to sudden death	• Principal component analysis and hierarchical clustering results showed that the expression patterns of the 20 CNS sites profiled were significantly different from all non-CNS tissues and were also similar to one another, indicating an underlying common expression signature<br>• The 20 sites could be segregated into discrete groups with underlying similarities in anatomical structure and, in many cases, functional activity
	GSE7307	Roth (2007)	• Normal and diseased human tissues representing over 90 distinct tissue types<br>• Patient death was due to sudden death	• The results of this study have not been publicly disclosed (only data are available)
