# Supplementary material for: Multi-study Integration of Brain Cancer Transcriptomes Reveals Organ-Level Molecular Signatures
Source: PLoS Comput Biol. 2013 Jul 25;9(7):e1003148. doi: 10.1371/journal.pcbi.1003148 (PMC3723500; doi:10.1371/journal.pcbi.1003148)
Supplement: Table S2 — GEO microarray sample IDs used in this study. (PDF) [file pcbi.1003148.s007.pdf]

Supplementary Table 2. GEO microarray sample IDs used in this study.

Phenotype Name	Phenotype Label	GEO Accession Series #	GEO Microarray Sample ID
Ependymoma	EPN	GSE16155	GSM404936,GSM404937,GSM404938,GSM404939,GSM404940,GSM404941,GSM404942,GSM404943,GSM404944,GSM404945,GSM404946,GSM404947,GSM404948,GSM404949,GSM404950,GSM404951,GSM404952,GSM404953,GSM404954
		GSE21687	GSM541060,GSM541061,GSM541062,GSM541063,GSM541064,GSM541065,GSM541066,GSM541067,GSM541068,GSM541069,GSM541070,GSM541071,GSM541072,GSM541073,GSM541074,GSM541075,GSM541076,GSM541077,GSM541078,GSM541079,GSM541080,GSM541081,GSM541082,GSM541083,GSM541084,GSM541085,GSM541086,GSM541087,GSM541088,GSM541089,GSM541090,GSM541091,GSM541092,GSM541093,GSM541094,GSM541095,GSM541096,GSM541097,GSM541098,GSM541099,GSM541100,GSM541101,GSM541102,GSM541103,GSM541104,GSM541105,GSM541106,GSM541107,GSM541108,GSM541109,GSM541110,GSM541111,GSM541112,GSM541113,GSM541114,GSM541115,GSM541116,GSM541117,GSM541118,GSM541119,GSM541120,GSM541121,GSM541122,GSM541123,GSM541124,GSM541125,GSM541126,GSM541127,GSM541128,GSM541129,GSM541130,GSM541131,GSM541132,GSM541133,GSM541134,GSM541135,GSM541136,GSM541137,GSM541138,GSM541139,GSM541140,GSM541141,GSM541142
Glioblastoma Multiforme	GBM	GSE 4412	GSM99432,GSM99434,GSM99436,GSM99438,GSM99440,GSM99442,GSM99444,GSM99446,GSM99448,GSM99450,GSM99452,GSM99454,GSM99456,GSM99462,GSM99464,GSM99466,GSM99470,GSM99472,GSM99474,GSM99476,GSM99478,GSM99480,GSM99482,GSM99484,GSM99486,GSM99488,GSM99490,GSM99492,GSM99494,GSM99524,GSM99526,GSM99528,GSM99530,GSM99532,GSM99534,GSM99536,GSM99538,GSM99540,GSM99542,GSM99544,GSM99546,GSM99548,GSM99550,GSM99552,GSM99554,GSM99556,GSM99558,GSM99560,GSM99562,GSM99564,GSM99572,GSM99576,GSM99578,GSM99580,GSM99582,GSM99584,GSM99586,GSM99588,GSM99590
		GSE 4271	GSM96950,GSM96951,GSM96952,GSM96953,GSM96954,GSM96955,GSM96956,GSM96957,GSM96958,GSM96959,GSM96960,GSM96961,GSM96962,GSM96963,GSM96964,GSM96965,GSM96966,GSM96967,GSM96968,GSM96969,GSM96970,GSM96971,GSM96972,GSM96973,GSM96974,GSM96975,GSM96976,GSM96977,GSM96978,GSM96979,GSM96980,GSM96981,GSM96982,GSM96983,GSM96984,GSM96985,GSM96986,GSM96987,GSM96988,GSM96989,GSM96990,GSM96991,GSM96992,GSM96993,GSM96994,GSM96995,GSM96996,GSM96997,GSM96998,GSM96999,GSM97000,GSM97001,GSM97002,GSM97003,GSM97004,GSM97005,GSM97006,GSM97007,GSM97008,GSM97009,GSM97010,GSM97011,GSM97014,GSM97018,GSM97021,GSM97024,GSM97028,GSM97031,GSM97032,GSM97037,GSM97040,GSM97041,GSM97042,GSM97044,GSM97048,GSM97049
		GSE 8692	GSM215420,GSM215422,GSM215423,GSM215425,GSM215426,GSM215427
		GSE 9171	GSM231695,GSM231696,GSM231697,GSM231698,GSM231699,GSM231700,GSM231701,GSM231702,GSM231703,GSM231704,GSM231705,GSM231706,GSM231707
		GSE 4290	GSM97794,GSM97796,GSM97797,GSM97798,GSM97801,GSM97806,GSM97808,GSM97813,GSM97814,GSM97818,GSM97819,GSM97821,GSM97829,GSM97832,GSM97839,GSM97844,GSM97847,GSM97851,GSM97852,GSM97856,GSM97859,GSM97861,GSM97863,GSM97869,GSM97870,GSM97871,GSM97877,GSM97882,GSM97885,GSM97886,GSM97887,GSM97888,GSM97889,GSM97891,GSM97892,GSM97893,GSM97894,GSM97895,GSM97896,GSM97898,GSM97903,GSM97905,GSM97906,GSM97908,GSM97912,GSM97914,GSM97915,GSM97917,GSM97918,GSM97919,GSM97922,GSM97924,GSM97926,GSM97930,GSM97931,GSM97935,GSM97936,GSM97938,GSM97940,GSM97942,GSM97945,GSM97946,GSM97948,GSM97950,GSM97952,GSM97953,GSM97954,GSM97955,GSM97959,GSM97961,GSM97963,GSM97965,GSM97966,GSM97967,GSM97968,GSM97969,GSM97971

Supplementary Table 2. (Continued) GEO microarray sample IDs used in this study.

Medulloblastoma	MDL	GSE 10327	GSM260959,GSM260960,GSM260961,GSM260962,GSM260963,GSM260964,GSM260965,GSM260966,GSM260967,GSM260968,GSM260969,GSM260970,GSM260971,GSM260972,GSM260973,GSM260974,GSM260975,GSM260976,GSM260977,GSM260978,GSM260979,GSM260981,GSM260982,GSM260983,GSM260984,GSM260985,GSM260986,GSM260987,GSM260988,GSM260989,GSM260990,GSM260991,GSM260992,GSM260993,GSM260994,GSM260995,GSM260996,GSM260997,GSM260998,GSM260999,GSM261000,GSM261001,GSM261002,GSM261003,GSM261004,GSM261005,GSM261006,GSM261007,GSM261008,GSM261009,GSM261010,GSM261011,GSM261012,GSM261013,GSM261014,GSM261015,GSM261016,GSM261017,GSM261018,GSM261019,GSM261020
		GSE 12992	GSM324062,GSM324063,GSM324064,GSM324065,GSM324066,GSM324067,GSM324068,GSM324069,GSM324082,GSM324083,GSM324084,GSM324085,GSM324090,GSM324091,GSM324092,GSM324093,GSM324104,GSM324111,GSM324112,GSM324113,GSM324115,GSM324119,GSM324137,GSM324138,GSM324139,GSM324140,GSM324141,GSM324508,GSM324512,GSM324513,GSM324514,GSM324515,GSM324516,GSM324517,GSM324526,GSM325233,GSM325278,GSM325280,GSM325281,GSM325282
Meningioma	MNG	GSE 4780	GSM108014,GSM108015,GSM108016,GSM108017,GSM108018,GSM107987,GSM107988,GSM107989,GSM107990,GSM107991,GSM107992,GSM107993,GSM107994,GSM107995,GSM107996,GSM107997,GSM107998,GSM107999,GSM108000,GSM108001,GSM108002,GSM108003,GSM108004,GSM108005,GSM108006,GSM108007,GSM108008,GSM108009,GSM108010,GSM108011,GSM108012,GSM108013,GSM108019,GSM108020,GSM108021,GSM108022,GSM108023,GSM108024,GSM108025,GSM108026,GSM108027,GSM108028,GSM108029,GSM108030,GSM108031,GSM108032,GSM108033,GSM108034,GSM108035,GSM108036,GSM108037,GSM108038,GSM108039,GSM108040,GSM108041,GSM108042,GSM108043,GSM108044,GSM108045,GSM108046,GSM108047,GSM108048
		GSE 9438	GSM239770,GSM239771,GSM239772,GSM239773,GSM239774,GSM239775,GSM239776,GSM239777,GSM239778,GSM239779,GSM239780,GSM239781,GSM239782,GSM239783,GSM239784,GSM239785,GSM239786,GSM239787,GSM239788,GSM239789,GSM239790,GSM239791,GSM239792,GSM239793,GSM239794,GSM239795,GSM239796,GSM239797,GSM239798,GSM239799,GSM239800
		GSE 16581	GSM416798,GSM416799,GSM416800,GSM416801,GSM416802,GSM416803,GSM416804,GSM416805,GSM416806,GSM416807,GSM416808,GSM416809,GSM416810,GSM416811,GSM416812,GSM416813,GSM416814,GSM416815,GSM416816,GSM416817,GSM416818,GSM416819,GSM416820,GSM416821,GSM416822,GSM416823,GSM416824,GSM416825,GSM416826,GSM416827,GSM416828,GSM416829,GSM416830,GSM416831,GSM416832,GSM416833,GSM416834,GSM416835,GSM416836,GSM416837,GSM416838,GSM416839,GSM416840,GSM416841,GSM416842,GSM416843,GSM416844,GSM416845,GSM416846,GSM416847,GSM416848,GSM416849,GSM416850,GSM416851,GSM416852,GSM416853,GSM416854,GSM416855,GSM416856,GSM416857,GSM416858,GSM416859,GSM416860,GSM416861,GSM416862,GSM416863,GSM416864,GSM416865
Oligodendroglioma	OLG	GSE 4412	GSM99458,GSM99460,GSM99510,GSM99512,GSM99514,GSM99516,GSM99518,GSM99520,GSM99522,GSM99570,GSM99598
		GSE 4290	GSM97799,GSM97822,GSM97823,GSM97824,GSM97830,GSM97831,GSM97835,GSM97838,GSM97841,GSM97842,GSM97845,GSM97854,GSM97857,GSM97860,GSM97862,GSM97864,GSM97865,GSM97866,GSM97867,GSM97868,GSM97872,GSM97873,GSM97874,GSM97875,GSM97876,GSM97880,GSM97881,GSM97883,GSM97884,GSM97897,GSM97900,GSM97901,GSM97902,GSM97904,GSM97907,GSM97909,GSM97911,GSM97923,GSM97925,GSM97928,GSM97929,GSM97933,GSM97934,GSM97944,GSM97947,GSM97949,GSM97956,GSM97962,GSM97964,GSM97970

Supplementary Table 2. (Continued) GEO microarray sample IDs used in this study.

Pilocytic Astrocytoma	PA	GSE 12907	GSM322969,GSM323054,GSM323523,GSM323524,GSM323525,GSM323526,GSM323527,GSM323528,GSM323529,GSM323530,GSM323531,GSM323554,GSM323555,GSM323557,GSM323558,GSM323559,GSM323560,GSM323561,GSM323562,GSM323563,GSM323564
		GSE 5675	GSM132714,GSM132715,GSM132716,GSM132717,GSM132718,GSM132719,GSM132720,GSM132722,GSM132723,GSM132728,GSM132729,GSM132730 ,GSM132733,GSM132736,GSM132738 ,GSM132741 ,GSM132744,GSM132747,GSM132748,GSM132750 ,GSM132751,GSM132752,GSM132753,GSM132754,GSM132759,GSM132761,GSM132763,GSM132765,GSM132768,GSM132769,GSM132770,GSM132771,GSM132772,GSM132773,GSM132774,GSM132775,GSM132776,GSM132777,GSM132778,GSM132779,GSM132780
Normal Brain	normal	GSE 3526	GSM80565,GSM80566,GSM80567,GSM80568,GSM80569,GSM80570,GSM80571,GSM80572,GSM80573,GSM80574,GSM80575,GSM80581,GSM80585,GSM80586,GSM80587,GSM80591,GSM80592,GSM80593,GSM80594,GSM80595,GSM80596,GSM80597,GSM80598,GSM80599,GSM80600,GSM80601,GSM80616,GSM80617,GSM80618,GSM80619,GSM80620,GSM80621,GSM80622,GSM80623,GSM80626,GSM80627,GSM80628,GSM80636,GSM80637,GSM80638,GSM80639,GSM80640,GSM80641,GSM80642,GSM80643,GSM80644,GSM80645,GSM80646,GSM80647,GSM80650,GSM80651,GSM80652,GSM80653,GSM80660,GSM80661,GSM80662,GSM80663,GSM80664,GSM80665,GSM80666,GSM80667,GSM80668,GSM80669,GSM80670,GSM80671,GSM80675,GSM80676,GSM80677,GSM80678,GSM80679,GSM80680,GSM80681,GSM80682,GSM80683,GSM80684,GSM80690,GSM80691,GSM80692,GSM80693,GSM80699,GSM80700,GSM80701,GSM80702,GSM80703,GSM80704,GSM80705,GSM80706,GSM80708,GSM80709,GSM80711,GSM80713,GSM80714,GSM80715,GSM80721,GSM80722,GSM80723,GSM80724,GSM80744,GSM80745,GSM80746,GSM80747,GSM80752,GSM80754,GSM80756,GSM80760,GSM80761,GSM80762,GSM80763,GSM80766,GSM80767,GSM80772,GSM80773,GSM80774,GSM80775,GSM80800,GSM80801,GSM80802,GSM80803,GSM80804,GSM80817,GSM80818,GSM80819,GSM80830,GSM80831,GSM80832,GSM80833,GSM80834,GSM80835,GSM80836,GSM80837,GSM80838,GSM80839,GSM80840,GSM80841,GSM80847,GSM80848,GSM80849,GSM80851,GSM80852,GSM80855,GSM80858,GSM80859,GSM80860,GSM80861,GSM80862,GSM80863
		GSE 7307	GSM175842,GSM175843,GSM175844,GSM175845,GSM175849,GSM175850,GSM175851,GSM176153,GSM175852,GSM175853,GSM175854,GSM176048,GSM175855,GSM175856,GSM175857,GSM175858,GSM175846,GSM175847,GSM175848,GSM176150,GSM175871,GSM175872,GSM175873,GSM176059,GSM175874,GSM175875,GSM175876,GSM175877,GSM176215,GSM175901,GSM175902,GSM175903,GSM175904,GSM175987,GSM175988,GSM176174,GSM176175,GSM175989,GSM175990,GSM176170,GSM176171,GSM176172,GSM176173,GSM176178,GSM176179,GSM176180,GSM176181,GSM176182,GSM176183,GSM176184,GSM176185,GSM176161,GSM176162,GSM176163,GSM176164,GSM176056,GSM176073
