# Supplementary material for: Multi-study Integration of Brain Cancer Transcriptomes Reveals Organ-Level Molecular Signatures
Source: PLoS Comput Biol. 2013 Jul 25;9(7):e1003148. doi: 10.1371/journal.pcbi.1003148 (PMC3723500; doi:10.1371/journal.pcbi.1003148)
Supplement: Table S3 — Node marker panel for brain cancer and normal transcriptome classification. Node #: Corresponds to numerical labels shown in the brain phenotype diagnostic hierarchy (Figure 1). Brain phenotype abbreviation (name): EPN (Ependymoma), GBM (Glioblastoma multiforme), MDL (Medulloblastoma), MNG (Meningioma), normal (Normal brain), OLG (Oligodendroglioma), and PA (Pilocytic astrocytoma). Gene i/Gene j: the gene expressed higher and lower in the gene-pair, respectively, within each corresponding phenotype. Gene name/Chromosome locus: according to Entrez Gene. Affymetrix Probe ID: For both Affymetrix Human Genome U133A and U133Plus2.0 Arrays. k: The minimum number of gene-pair classifiers whose decision rule outcomes for a test sample are required to be ‘true ( = 1)’ for the sample to be classified as the phenotype(s) of the corresponding node. (PDF) [file pcbi.1003148.s008.pdf]

Table S3. Node marker panel for brain cancer and normal transcriptome classification.

Node #	Node phenotype classes	Gene *i*				Gene *j*				*k*
		Gene symbol	Gene name	Chomosome locus	Affymetrix Probe ID	Gene symbol	Gene name	Chomosome locus	Affymetrix Probe ID	
2	EPN GBM MDL MNG OLG PA	*PRPF40A*	PRP40 pre-mRNA processing factor 40 homolog A (S. cerevisiae)	2q23.3	218053_at	*PURA*	Purine-rich element binding protein A	5q31	204021_s_at	1
3	normal	*PURA*	Purine-rich element binding protein A	5q31	204021_s_at	*PRPF40A*	PRP40 pre-mRNA processing factor 40 homolog A (S. cerevisiae)	2q23.3	218053_at	1
4	EPN GBM MDL OLG PA	*NRCAM*	Neuronal cell adhesion molecule	7q31	204105_s_at	*ISLR*	Immunoglobulin superfamily containing leucine-rich repeat	15q23-q24	207191_s_at	1
		IDH2	Isocitrate dehydrogenase 2 (NADP+), mitochondrial	15q26.1	210046_s_at	*GMDS*	GDP-mannose 4,6-dehydratase	6p25	214106_s_at	
5	MNG	*ISLR*	Immunoglobulin superfamily containing leucine-rich repeat	15q23-q24	207191_s_at	*NRCAM*	Neuronal cell adhesion molecule	7q31	204105_s_at	1
6	EPN GBM OLG PA	*SALL1*	Sal-like 1 (Drosophila)	16q12.1	206893_at	*PAFAH1B3*	Platelet-activating factor acetylhydrolase 1b, catalytic subunit 3	19q13.1	203228_at	2
		SRI	Sorcin	7q21	208920_at	*NBEA*	Neurobeachin	13q13	221207_s_at	
		DDR1	Discoidin domain receptor tyrosine kinase 1	6p21.3	210749_x_at	*TIA1*	TIA1 cytotoxic granule-associated RNA binding protein	2p13	201447_at	
		DDR1	Discoidin domain receptor tyrosine kinase 1	6p21.3	208779_x_at	*MAB21L1*	Mab-21-like 1 (C. elegans)	13q13	206163_at	
		ITPKB	Inositol 1,4,5-trisphosphate 3-kinase B	1q42.13	203723_at	*PDS5B*	PDS5, regulator of cohesion maintenance, homolog B (S. cerevisiae	13q12.3	204742_s_at	
7	MDL	*PAFAH1B3*	Platelet-activating factor acetylhydrolase 1b, catalytic subunit 3	19q13.1	203228_at	*SALL1*	Sal-like 1 (Drosophila)	16q12.1	206893_at	4
		NBEA	Neurobeachin	13q13	221207_s_at	*SRI*	Sorcin	7q21	208920_at	
		TIA1	TIA1 cytotoxic granule-associated RNA binding protein	2p13	201447_at	*DDR1*	Discoidin domain receptor tyrosine kinase 1	6p21.3	210749_x_at	
		MAB21L1	Mab-21-like 1 (C. elegans)	13q13	206163_at	*DDR1*	Discoidin domain receptor tyrosine kinase 1	6p21.3	208779_x_at	
		PDS5B	PDS5, regulator of cohesion maintenance, homolog B (S. cerevisiae	13q12.3	204742_s_at	*ITPKB*	Inositol 1,4,5-trisphosphate 3-kinase B	1q42.13	203723_at	
8	EPN	*NUP62CL*	Nucleoporin 62kDa C-terminal like	Xq22.3	220520_s_at	*ZNF280A*	Zinc finger protein 280A	22q11.22	216034_at	2
		GALNS	Galactosamine (N-acetyl)-6-sulfate sulfatase	16q24.3	206335_at	*WAS*	Wiskott-Aldrich syndrome (eczema-thrombocytopenia)	Xp11.4-p11.21	38964_r_at	
		CELSR1	Cadherin, EGF LAG seven-pass G-type receptor 1 (flamingo homolog, Drosophila)	22q13.3	41660_at	*OR10H3*	Olfactory receptor, family 10, subfamily H, member 3	19p13.1	208520_at	
		TLE4	Transducin-like enhancer of split 4 (E(sp1) homolog, Drosophila)	9q21.31	216997_x_at	*OLIG2*	Oligodendrocyte lineage transcription factor 2	21q22.11	213824_at	

Table S3. (Continued) Node marker panel for brain cancer and normal transcriptome classification.

Node #	Node phenotype classes	Gene *i*				Gene *j*				*k*
		Gene symbol	Gene name	Chomosome locus	Affymetrix Probe ID	Gene symbol	Gene name	Chomosome locus	Affymetrix Probe ID	
9	GBM OLG PA	*ZNF280A*	Zinc finger protein 280A	22q11.22	216034_at	*NUP62CL*	Nucleoporin 62kDa C-terminal like	Xq22.3	220520_s_at	1
10	GBM OLG	*DDX27*	DEAD (Asp-Glu-Ala-Asp) box polypeptide 27	20q13.13	215693_x_at	*KCNMA1*	Potassium large conductance calcium-activated channel, subfamily M, alpha member 1	10q22.3	221584_s_at	1
		COX7A2	Cytochrome c oxidase subunit VIIa polypeptide 2 (liver)	6q12	217249_x_at	*GNPTAB*	N-acetylglucosamine-1-phosphate transferase, alpha and beta subunits	12q23.2	212959_s_at	
11	PA	*KCNMA1*	Potassium large conductance calcium-activated channel, subfamily M, alpha member 1	10q22.3	221584_s_at	*DDX27*	DEAD (Asp-Glu-Ala-Asp) box polypeptide 27	20q13.13	215693_x_at	3
		GNPTAB	N-acetylglucosamine-1-phosphate transferase, alpha and beta subunits	12q23.2	212959_s_at	*NDUFS2*	NADH dehydrogenase (ubiquinone) Fe-S protein 2, 49kDa (NADH-coenzyme Q reductase)	1q23	201966_at	
		APOD	Apolipoprotein D	3q26.2-qter	201525_at	*PPIA*	Peptidylprolyl isomerase A (cyclophilin A)	7p13	211378_x_at	
		CD59	CD59 molecule, complement regulatory protein	11p13	212463_at	*SNRPB2*	Small nuclear ribonucleoprotein polypeptide B	20p12.1	202505_at	
		SEMA3E	Sema domain, immunoglobulin domain (Ig), short basic domain, secreted, (semaphorin) 3E	7q21.11	206941_x_at	*ADAMTS3*	ADAM metallopeptidase with thrombospondin type 1 motif, 3	4q13.3	214913_at	
		CD59	CD59 molecule, complement regulatory protein	11p13	200985_s_at	*HINT1*	Histidine triad nucleotide binding protein 1	5q31.2	208826_x_at	
		BAMBI	BMP and activin membrane-bound inhibitor homolog (Xenopus laevis)	10p12.13-p11.2	203304_at	*CIAPIN1*	Cytokine induced apoptosis inhibitor 1	16q13-q21	208968_s_at	
12	GBM	*FLNA*	Filamin A, alpha	Xq28	214752_x_at	*TNKS2*	Tankyrase, TRF1-interacting ankyrin-related ADP-ribose polymerase 2	10q23.3	218228_s_at	1
		ITGB3BP	Integrin beta 3 binding protein (beta3-endonexin)	1p31.3	205176_s_at	*RB1CC1*	RB1-inducible coiled-coil 1	8q11	202034_x_at	
		DDX27	DEAD (Asp-Glu-Ala-Asp) box polypeptide 27	20q13.13	215693_x_at	*TRIM8*	Tripartite motif-containing 8	10q24.3	221012_s_at	
13	OLG	*LARP5*	La ribonucleoprotein domain family, member 4B	10p15.3	208953_at	*ANXA1*	Annexin A1	9q12-q21.2	201012_at	1

Node #: Corresponds to numerical labels shown in the brain phenotype diagnostic hierarchy (**Fig. 1**). **Brain phenotype abbreviation (name):** EPN (Ependymoma), GBM (Glioblastoma multiforme), MDL (Medulloblastoma), MNG (Meningioma), normal (Normal brain), OLG (Oligodendroglioma), and PA (Pilocytic astrocytoma). **Gene *i* / Gene *j*:** the gene expressed higher and lower in the gene-pair, respectively, within each corresponding phenotype. **Gene name / Chromosome locus:** according to Entrez Gene. **Affymetrix Probe ID:** For both Affymetrix Human Genome U133A and U133Plus2.0 Arrays. ***k*:** The minimum number of gene-pair classifiers whose decision rule outcomes for a test sample are required to be 'true (= 1)' for the sample to be classified as the phenotype(s) of the corresponding node.
