# Supplementary material for: Multi-study Integration of Brain Cancer Transcriptomes Reveals Organ-Level Molecular Signatures
Source: PLoS Comput Biol. 2013 Jul 25;9(7):e1003148. doi: 10.1371/journal.pcbi.1003148 (PMC3723500; doi:10.1371/journal.pcbi.1003148)
Supplement: Table S4 — Decision-tree marker panel for brain cancer and normal transcriptome classification. For each classifier decision rule (i.e. Is Gene i>Gene j ?), 1 and 0 delineates ‘true’ and ‘false’, respectively, and ‘–’ denotes that the outcome is not used for classification. The vertical binary pattern under each class label corresponds to a phenotype-specific molecular signature. (PDF) [file pcbi.1003148.s009.pdf]

Table S4. Decision-tree marker panel for brain cancer and normal transcriptome classification.

Gene *i*		Gene *j*		Brain phenotype binary signature						
Gene symbol	Affymetrix Probe ID	Gene symbol	Affymetrix Probe ID	EPN	GBM	MDL	MNG	OLG	PA	normal
PRPF40A	218053_at	*PURA*	204021_s_at	1	1	1	1	1	1	0
NRCAM	204105_s_at	*ISLR*	207191_s_at	1	1	1	0	1	1	-
SRI	208920_at	*NBEA*	221207_s_at	1	1	0	-	1	1	-
NUP62CL	220520_s_at	*OR10H3*	208520_at	1	0	-	-	0	0	-
DDX27	215693_x_at	*KCNMA1*	221584_s_at	-	1	-	-	1	0	-
FLNA	214752_x_at	*TNKS2*	218228_s_at	-	1	-	-	0	-	-

For each classifier decision rule (i.e. Is Gene *i* > Gene *j* ?), 1 and 0 delineates 'true' and 'false', respectively, and ' – ' denotes that the outcome is not used for classification. The vertical binary pattern under each class label corresponds to a phenotype-specific molecular signature.
