# Supplementary material for: Multi-study Integration of Brain Cancer Transcriptomes Reveals Organ-Level Molecular Signatures
Source: PLoS Comput Biol. 2013 Jul 25;9(7):e1003148. doi: 10.1371/journal.pcbi.1003148 (PMC3723500; doi:10.1371/journal.pcbi.1003148)
Supplement: Table S5 — Summary of expression differences between genes-pair classifiers. Node #: Corresponds to numerical labels shown in the brain phenotype diagnostic hierarchy (Figure 1). Brain phenotype abbreviation (name): EPN (Ependymoma), GBM (Glioblastoma multiforme), MDL (Medulloblastoma), MNG (Meningioma), normal (Normal brain), OLG (Oligodendroglioma), and PA (Pilocytic astrocytoma). Sample number: Number of total samples in classes of respective Node #. Gene i/Gene j: the gene expressed higher and lower in the gene-pair, respectively, within each corresponding phenotype. Gene name/Chromosome locus: according to Entrez Gene. Affymetrix Probe ID: For both Affymetrix Human Genome U133A and U133Plus2.0 Arrays. k: The minimum number of gene-pair classifiers whose decision rule outcomes for a test sample are required to be ‘true ( = 1)’ for the sample to be classified as the phenotype(s) of the corresponding node. Ranked expression differences of each gene pair (i.e. Rank_gene_i – Rank_gene_j) were calculated for each sample, and Mean, St. dev., Max., Min., and Median were found across all samples within classes of respective Node #. (PDF) [file pcbi.1003148.s010.pdf]

Table S5. Summary of expression differences between genes-pair classifiers.

Node #	Node phenotype classes	Sample number	Gene *i*		Gene *j*		*k*	Mean	St. dev.	Max.	Min.	Median
			Gene symbol	Affymetrix Probe ID	Gene symbol	Affymetrix Probe ID						
2	EPN GBM MDL MNG OLG PA	718	*PRPF40A*	218053_at	*PURA*	204021_s_at	1	4,314.7	1,685.5	10,889.0	-2,593.0	4,370.5
3	normal	203	*PURA*	204021_s_at	*PRPF40A*	218053_at	1	1,793.5	1,122.2	9,249.0	176.0	1,591.0
4	EPN GBM MDL OLG PA	557	*NRCAM*	204105_s_at	*ISLR*	207191_s_at	1	10,376.0	2,475.5	13,271.0	-7,491.0	11,283.0
			IDH2	210046_s_at	*GMDS*	214106_s_at		5,153.1	1,647.1	9,264.0	-6,662.0	5,238.0
5	MNG	161	*ISLR*	207191_s_at	*NRCAM*	204105_s_at	1	8,568.9	2,449.9	11,884.0	-11,674.0	8,977.0
6	EPN GBM OLG PA	456	*SALL1*	206893_at	*PAFAH1B3*	203228_at	2	8,239.6	2,767.1	12,574.0	-9,364.0	8,728.0
			SRI	208920_at	*NBEA*	221207_s_at		4,513.8	2,001.8	10,692.0	-401.0	4,536.5
			DDR1	210749_x_at	*TIA1*	201447_at		4,227.7	1,528.7	9,580.0	-3,227.0	4,536.0
			DDR1	208779_x_at	*MAB21L1*	206163_at		9,514.9	2,223.0	13,251.0	-1,759.0	10,267.0
			ITPKB	203723_at	*PDS5B*	204742_s_at		7,051.7	2,551.4	11,373.0	-1,524.0	7,478.5
7	MDL	101	*PAFAH1B3*	203228_at	*SALL1*	206893_at	4	8,729.6	2,776.6	12,096.0	9.0	9,341.0
			NBEA	221207_s_at	*SRI*	208920_at		3,858.3	2,074.7	8,440.0	-28.0	3,715.0
			TIA1	201447_at	*DDR1*	210749_x_at		3,476.4	1,618.5	8,709.0	27.0	3,481.0
			MAB21L1	206163_at	*DDR1*	208779_x_at		4,510.6	1,845.9	8,771.0	-5,415.0	4,562.0
			PDS5B	204742_s_at	*ITPKB*	203723_at		5,495.4	2,515.1	11,625.0	-2,026.0	5,538.0
8	EPN	102	*NUP62CL*	220520_s_at	*ZNF280A*	216034_at	2	5,676.0	3,444.6	11,512.0	-830.0	6,079.5
			GALNS	206335_at	*WAS*	38964_r_at		3,952.7	2,095.5	7,831.0	-1,216.0	4,098.5
			CELSR1	41660_at	*OR10H3*	208520_at		4,959.8	2,798.6	11,365.0	-1,367.0	5,058.5
			TLE4	216997_x_at	*OLIG2*	213824_at		3,423.4	2,401.5	9,765.0	-3,239.0	3,386.5

Node #: Corresponds to numerical labels shown in the brain phenotype diagnostic hierarchy (**Fig. 1**). **Brain phenotype abbreviation (name):** EPN (Ependymoma), GBM (Glioblastoma multiforme), MDL (Medulloblastoma), MNG (Meningioma), normal (Normal brain), OLG (Oligodendroglioma), and PA (Pilocytic astrocytoma). **Sample number:** Number of total samples in classes of respective Node #. **Gene *i* / Gene *j*:** the gene expressed higher and lower in the gene-pair, respectively, within each corresponding phenotype. **Gene name / Chromosome locus:** according to Entrez Gene. **Affymetrix Probe ID:** For both Affymetrix Human Genome U133A and U133Plus2.0 Arrays. ***k*:** The minimum number of gene-pair classifiers whose decision rule outcomes for a test sample are required to be 'true (= 1)' for the sample to be classified as the phenotype(s) of the corresponding node. Ranked expression differences of each gene pair (i.e. Rank_gene_*i* – Rank_gene_*j*) were calculated for each sample, and **Mean, St. dev., Max., Min.,** and **Median** were found across all samples within classes of respective Node #.

Table S5. (Continued) Summary of expression differences between genes-pair classifiers.

Node #	Node phenotype classes	Sample number	Gene *i*		Gene *j*		*k*	Mean	St. dev.	Max.	Min.	Median
			Gene symbol	Affymetrix Probe ID	Gene symbol	Affymetrix Probe ID						
9	GBM OLG PA	354	*ZNF280A*	216034_at	*NUP62CL*	220520_s_at	1	1,392.5	673.9	3,211.0	-4,279.0	1,354.0
10	GBM OLG	292	*DDX27*	215693_x_at	*KCNMA1*	221584_s_at	1	2,144.8	1,366.3	6,451.0	-1,681.0	2,124.5
			COX7A2	217249_x_at	*GNPTAB*	212959_s_at		1,777.2	1,106.8	5,125.0	-2,040.0	1,664.0
11	PA	62	*KCNMA1*	221584_s_at	*DDX27*	215693_x_at	3	1,832.2	942.5	3,558.0	105.0	1,853.0
			GNPTAB	212959_s_at	*NDUFS2*	201966_at		1,149.7	737.3	3,061.0	-825.0	1,112.5
			APOD	201525_at	*PPIA*	211378_x_at		121.4	117.3	494.0	-426.0	104.5
			CD59	212463_at	*SNRPB2*	202505_at		1,169.1	1,038.0	3,989.0	-2,247.0	1,059.0
			SEMA3E	206941_x_at	*ADAMTS3*	214913_at		4,376.3	3,026.1	8,551.0	-8,061.0	5,194.5
			CD59	200985_s_at	*HINT1*	208826_x_at		388.5	422.5	1,586.0	-270.0	214.5
			BAMBI	203304_at	*CIAPIN1*	208968_s_at		1,976.4	975.1	3,802.0	-594.0	1,984.0
12	GBM	231	*FLNA*	214752_x_at	*TNKS2*	218228_s_at	1	3,238.8	2,804.3	9,691.0	-4,133.0	3,557.0
			ITGB3BP	205176_s_at	*RB1CC1*	202034_x_at		1,793.7	2,357.9	8,665.0	-4,597.0	1,560.0
			DDX27	215693_x_at	*TRIM8*	221012_s_at		1,819.1	1,492.6	6,083.0	-1,099.0	1,636.0
13	OLG	61	*LARP5*	208953_at	*ANXA1*	201012_at	1	2,843.4	3,463.0	9,055.0	-5,831.0	2,788.0

Node #: Corresponds to numerical labels shown in the brain phenotype diagnostic hierarchy (**Fig. 1**). **Brain phenotype abbreviation (name):** EPN (Ependymoma), GBM (Glioblastoma multiforme), MDL (Medulloblastoma), MNG (Meningioma), normal (Normal brain), OLG (Oligodendroglioma), and PA (Pilocytic astrocytoma). **Sample number:** Number of total samples in classes of respective Node #. **Gene *i* / Gene *j*:** the gene expressed higher and lower in the gene-pair, respectively, within each corresponding phenotype. **Gene name / Chromosome locus:** according to Entrez Gene. **Affymetrix Probe ID:** For both Affymetrix Human Genome U133A and U133Plus2.0 Arrays. ***k*:** The minimum number of gene-pair classifiers whose decision rule outcomes for a test sample are required to be 'true (= 1)' for the sample to be classified as the phenotype(s) of the corresponding node. Ranked expression differences of each gene pair (i.e. Rank_gene_*i* – Rank_gene_*j*) were calculated for each sample, and **Mean, St. dev., Max., Min.,** and **Median** were found across all samples within classes of respective Node #.
