# Supplementary material for: Multi-study Integration of Brain Cancer Transcriptomes Reveals Organ-Level Molecular Signatures
Source: PLoS Comput Biol. 2013 Jul 25;9(7):e1003148. doi: 10.1371/journal.pcbi.1003148 (PMC3723500; doi:10.1371/journal.pcbi.1003148)
Supplement: Table S6 — Hold-one-lab-in validation accuracies of glioblastoma signatures. (PDF) [file pcbi.1003148.s011.pdf]

Table S6. Hold-one-lab-in validation accuracies of glioblastoma signatures.

GBM training set (sample size)	GBM test set (sample size)	Predicted phenotype / % of test set / samples of test set

GBM training set: GSE4412 (59)

GBM test set: GSE4271 (76)

UC	EPN	GBM	MDL	MNG	OLG	PA	Total
2.63%	57.89%	**9.21%**	17.11%	5.26%	1.32%	6.58%	76
2	44	7	13	4	1	5	76

GBM test set: GSE8692 (6)

GBM	MNG	Total
**83.33%**	16.67%	6
5	1	6

GBM test set: GSE9171 (13)

EPN	GBM	MNG	Total
92.31%	**0.00%**	7.69%	13
12	0	1	13

GBM test set: GSE4290 (77)

EPN	GBM	MDL	MNG	PA	Total
85.71%	**0.00%**	2.60%	5.19%	6.49%	77
66	0	2	4	5	77

GBM training set: GSE4271 (76)

GBM test set: GSE4412 (59)

UC	GBM	PA	normal	Total
11.86%	**77.97%**	8.47%	1.69%	59
7	46	5	1	59

GBM test set: GSE8692 (6)

GBM	Total
**100.0%**	6
6	6

GBM test set: GSE9171 (13)

GBM	6	Total
**92.31%**	7.69%	13
12	1	13

GBM test set: GSE4290 (77)

UC	GBM	MNG	PA	Total
5.19%	**77.92%**	1.30%	15.58%	77
4	60	1	12	77

GBM training set: GSE8692 (6)

GBM test set: GSE4412 (59)

UC	EPN	GBM	MDL	MNG	PA	normal	Total
5.08%	13.56%	**47.46%**	1.69%	3.39%	27.12%	1.69%	59
3	8	28	1	2	16	1	59

GBM test set: GSE4271 (76)

UC	EPN	GBM	MDL	PA	normal	Total
9.21%	32.89%	**18.42%**	5.26%	32.89%	1.32%	76
7	25	14	4	25	1	76

GBM test set: GSE9171 (13)

EPN	GBM	MDL	PA	Total
61.54%	**15.38%**	15.38%	7.69%	13
8	2	2	1	13

GBM test set: GSE4290 (77)

UC	EPN	GBM	MDL	MNG	PA	normal	Total
14.29%	42.86%	**7.79%**	1.30%	1.30%	25.97%	6.49%	77
11	33	6	1	1	20	5	77

Table S6. (Continued) Hold-one-lab-in validation accuracies of glioblastoma signatures.

GSE9171 (13)

GSE4412 (59)

UC	EPN	GBM	MDL	MNG	PA	Total
35.59%	13.56%	**0.00%**	1.69%	5.08%	44.07%	59
21	8	0	1	3	26	59

GSE4271 (76)

UC	EPN	GBM	MDL	MNG	PA	Total
19.74%	38.16%	**0.00%**	6.58%	3.95%	31.58%	76
15	29	0	5	3	24	76

GSE8692 (6)

UC	GBM	MNG	PA	Total
66.67%	**0.00%**	16.67%	16.67%	6
4	0	1	1	6

GSE4290 (77)

UC	EPN	GBM	MDL	PA	normal	Total
10.39%	40.26%	**0.00%**	1.30%	46.75%	1.30%	77
8	31	0	1	36	1	77

GSE4290 (77)

GSE4412 (59)

UC	GBM	NB	PA	normal	Total
5.08%	**52.54%**	27.12%	13.56%	1.69%	59
3	31	16	8	1	59

GSE4271 (76)

UC	EPN	GBM	MDL	OLG	PA	Total
1.32%	1.32%	**60.53%**	3.95%	15.79%	17.11%	76
1	1	46	3	12	13	76

GSE8692 (6)

UC	GBM	NB	PA	Total
33.33%	**16.67%**	16.67%	33.33%	6
2	1	1	2	6

GSE9171 (13)

UC	GBM	Total
7.69%	**92.31%**	13
1	12	13
