# Supplementary material for: Multi-study Integration of Brain Cancer Transcriptomes Reveals Organ-Level Molecular Signatures
Source: PLoS Comput Biol. 2013 Jul 25;9(7):e1003148. doi: 10.1371/journal.pcbi.1003148 (PMC3723500; doi:10.1371/journal.pcbi.1003148)
Supplement: Table S7 — Hold-one-lab-in (H1LI) and leave-one-lab-out (L1LO) validation accuracies of glioblastoma signatures when training data were constrained to 50 total samples. H1LI and L1LO validations were performed ten times for each category of training data. In each validation trial, 50 samples were randomly selected from the single microarray dataset (for H1L1) or from the multi-study, combined dataset (for L1LO). (PDF) [file pcbi.1003148.s012.pdf]

Table S7. Hold-one-lab-in (H1LI) and leave-one-lab-out (L1LO) validation accuracies of glioblastoma signatures when training data were constrained to 50 total samples.

Method	GBM training set (50 samples)	GBM test set	GBM prediction		Average performance
			Average accuracy	St. dev.	
H1LI	GSE4412	GSE4271	40.26%	14.98%	36.39%
		GSE8692	96.67%	7.03%	
		GSE9171	6.15%	3.24%	
		GSE4290	2.47%	2.10%	
	GSE4271	GSE4412	58.98%	21.64%	63.89%
		GSE8692	74.00%	11.43%	
		GSE9171	73.08%	10.41%	
		GSE4290	49.48%	26.56%	
	GSE4290	GSE4412	38.47%	10.23%	40.66%
		GSE4271	43.13%	16.12%	
		GSE8692	23.33%	9.08%	
		GSE9171	57.70%	16.79%	
L1LO	GSE4271, GSE8692, GSE9171, GSE4290	GSE4412	82.20%	10.39%	69.72%
	GSE4412, GSE8692, GSE9171, GSE4290	GSE4271	54.87%	7.18%	
	GSE4412, GSE4271, GSE8692, GSE9171	GSE4290	72.08%	15.29%	

H1LI and L1LO validations were performed ten times for each category of training data. In each validation trial, 50 samples were randomly selected from the single microarray dataset (for H1L1) or from the multi-study, combined dataset (for L1LO).
