# Supplementary material for: Multi-study Integration of Brain Cancer Transcriptomes Reveals Organ-Level Molecular Signatures
Source: PLoS Comput Biol. 2013 Jul 25;9(7):e1003148. doi: 10.1371/journal.pcbi.1003148 (PMC3723500; doi:10.1371/journal.pcbi.1003148)
Supplement: Table S8 — Ten-fold cross-validation accuracies when only the node marker panel was required to reach unique diagnoses. Sample size: Average proportion of total samples that reached unique diagnoses via node marker panel. Accuracy: Reflects average performance in ten-fold cross-validation conducted ten times. (PDF) [file pcbi.1003148.s013.pdf]

Table S8. Ten-fold cross-validation accuracies when only the node marker panel was required to reach unique diagnoses.

Phenotype	Total samples	Sample size (%)	Accuracy (%)
EPN	102	93.1	95.8
GBM	231	88.9	92.7
MDL	101	95.0	95.8
MNG	161	98.8	97.5
OLG	61	77.0	74.5
PA	62	90.3	96.4
Normal	203	97.9	99.5
Average	-	91.6	93.2

Sample size: Average proportion of total samples that reached unique diagnoses via node marker panel. **Accuracy:** Reflects average performance in ten-fold cross-validation conducted ten times.
