# Supplementary material for: Multi-study Integration of Brain Cancer Transcriptomes Reveals Organ-Level Molecular Signatures
Source: PLoS Comput Biol. 2013 Jul 25;9(7):e1003148. doi: 10.1371/journal.pcbi.1003148 (PMC3723500; doi:10.1371/journal.pcbi.1003148)
Supplement: Table S9 — Functional roles of 11 previously identified GBM serum markers that are present in our extracellular-product encoding marker-panel. a http://www.ncbi.nlm.nih.gov/gene. (PDF) [file pcbi.1003148.s014.pdf]

Table S9. Functional roles of 11 previously identified GBM serum markers that are present in our extracellular-product encoding marker-panel.

Gene	Gene name	Functional role[a]
APOD	Apolipoprotein D	This gene encodes a component of high density lipoprotein that has no marked similarity to other apolipoprotein sequences. It has a high degree of homology to plasma retinol-binding protein and other members of the alpha 2 microglobulin protein superfamily of carrier proteins, also known as lipocalins. This glycoprotein is closely associated with the enzyme lecithin:cholesterol acyltransferase - an enzyme involved in lipoprotein metabolism.
CALU	Calumenin	The product of this gene is a calcium-binding protein localized in the endoplasmic reticulum (ER) and it is involved in such ER functions as protein folding and sorting. This protein belongs to a family of multiple EF-hand proteins (CERC) that include reticulocalbin, ERC-55, and Cab45 and the product of this gene. Alternatively spliced transcript variants encoding different isoforms have been identified.
CD163	Cluster of Differentiation 163	The protein encoded by this gene is a member of the scavenger receptor cysteine-rich (SRCR) superfamily, and is exclusively expressed in monocytes and macrophages. It functions as an acute phase-regulated receptor involved in the clearance and endocytosis of hemoglobin/haptoglobin complexes by macrophages, and may thereby protect tissues from free hemoglobin-mediated oxidative damage. This protein may also function as an innate immune sensor for bacteria and inducer of local inflammation. Alternatively spliced transcript variants encoding different isoforms have been described for this gene.
CHI3L1	Chitinase-3-like protein 1	Chitinases catalyze the hydrolysis of chitin, which is an abundant glycopolymer found in insect exoskeletons and fungal cell walls. The glycoside hydrolase 18 family of chitinases includes eight human family members. This gene encodes a glycoprotein member of the glycosyl hydrolase 18 family. The protein lacks chitinase activity and is secreted by activated macrophages, chondrocytes, neutrophils and synovial cells. The protein is thought to play a role in the process of inflammation and tissue remodeling.
CSF1	Colony stimulating factor 1	The protein encoded by this gene is a cytokine that controls the production, differentiation, and function of macrophages. The active form of the protein is found extracellularly as a disulfide-linked homodimer, and is thought to be produced by proteolytic cleavage of membrane-bound precursors. The encoded protein may be involved in development of the placenta. Alternate splicing results in multiple transcript variants.
EGFR	Epidermal growth factor receptor	The protein encoded by this gene is a transmembrane glycoprotein that is a member of the protein kinase superfamily. This protein is a receptor for members of the epidermal growth factor family. EGFR is a cell surface protein that binds to epidermal growth factor. Binding of the protein to a ligand induces receptor dimerization and tyrosine autophosphorylation and leads to cell proliferation. Mutations in this gene are associated with lung cancer. Multiple alternatively spliced transcript variants that encode different protein isoforms have been found for this gene.
IGFBP2	Insulin-like growth factor binding protein 2	Inhibits IGF-mediated growth and developmental rates. IGF-binding proteins prolong the half-life of the IGFs and have been shown to either inhibit or stimulate the growth promoting effects of the IGFs on cell culture. They alter the interaction of IGFs with their cell surface receptors.
NID1	Nidogen 1	This gene encodes a member of the nidogen family of basement membrane glycoproteins. The protein interacts with several other components of basement membranes, and may play a role in cell interactions with the extracellular matrix.
PDGFC	Platelet derived growth factor C	The protein encoded by this gene is a member of the platelet-derived growth factor family. The four members of this family are mitogenic factors for cells of mesenchymal origin and are characterized by a core motif of eight cysteines. This gene product appears to form only homodimers. It differs from the platelet-derived growth factor alpha and beta polypeptides in having an unusual N-terminal domain, the CUB domain. Alternatively spliced transcript variants have been found for this gene.
PSG9	Pregnancy specific beta-1-glycoprotein 9	The human pregnancy-specific glycoproteins (PSGs) are a group of molecules that are mainly produced by the placental syncytiotrophoblasts during pregnancy. PSGs comprise a subgroup of the carcinoembryonic antigen (CEA) family, which belongs to the immunoglobulin superfamily.
PTN	Pleiotrophin	Secreted growth factor that induces neurite outgrowth and which is mitogenic for fibroblasts, epithelial, and endothelial cells. Binds anaplastic lymphoma kinase (ALK) which induces MAPK pathway activation, an important step in the anti-apoptotic signaling of PTN and regulation of cell proliferation

[a]http://www.ncbi.nlm.nih.gov/gene
