# Supplementary material for: Multi-study Integration of Brain Cancer Transcriptomes Reveals Organ-Level Molecular Signatures
Source: PLoS Comput Biol. 2013 Jul 25;9(7):e1003148. doi: 10.1371/journal.pcbi.1003148 (PMC3723500; doi:10.1371/journal.pcbi.1003148)
Supplement: Table S10 — Ten-fold cross-validation accuracies of gene-pair classifiers composed of genes that encode extracellular products. aAccuracies reflect average performance in ten-fold cross-validation conducted ten times. The main diagonal gives the average classification accuracy of each class (bold), and the off-diagonal elements show the erroneous predictions. bUC (Unclassified samples). When using the node classifiers, expression profiles that did not exert a signature of any phenotype (i.e., did not percolate down to at least one positive terminal node) were rejected from classification. In this case, the Unclassified sample is treated as a misclassification. (PDF) [file pcbi.1003148.s015.pdf]

Table S10. Ten-fold cross-validation accuracies of gene-pair classifiers composed of genes that encode extracellular products.

		Predicted phenotype (%)[a]								
		EPN	GBM	MDL	MNG	OLG	PA	normal	UC[b]	total
Actual phenotype	EPN	**83.3**	2.9	4.9	4.9	2.0	1.0	0.0	1.0	102
	GBM	2.2	**72.3**	0.4	1.3	20.3	0.9	0.0	2.6	231
	MDL	1.0	1.0	**94.1**	1.0	2.0	0.0	0.0	1.0	101
	MNG	0.0	1.2	0.6	**97.5**	0.0	0.0	0.0	0.6	161
	OLG	0.0	16.4	0.0	0.0	**75.4**	3.3	1.6	3.3	61
	PA	1.6	1.6	0.0	0.0	3.2	**91.9**	0.0	1.6	62
	normal	0.0	1.0	0.0	0.0	1.5	0.0	**96.6**	1.0	203

[a]Accuracies reflect average performance in ten-fold cross-validation conducted ten times. The main diagonal gives the average classification accuracy of each class (bold), and the off-diagonal elements show the erroneous predictions.
[b]UC (Unclassified samples). When using the node classifiers, expression profiles that did not exert a signature of any phenotype (i.e., did not percolate down to at least one positive terminal node) were rejected from classification. In this case, the Unclassified sample is treated as a misclassification.
