# Supplementary material for: Multi-study Integration of Brain Cancer Transcriptomes Reveals Organ-Level Molecular Signatures
Source: PLoS Comput Biol. 2013 Jul 25;9(7):e1003148. doi: 10.1371/journal.pcbi.1003148 (PMC3723500; doi:10.1371/journal.pcbi.1003148)
Supplement: Text S1 — Step-by-step description of how ISSAC works. (PDF) [file pcbi.1003148.s016.pdf]

Text S1. Step-by-step description of how ISSAC works

a. Construction of the disease diagnostic hierarchy

Let L $= (d_1, \dots, d_7)$ be the collection of class labels, where d_i denotes brain phenotype i. Using expression profiles of the phenotype classes, we first calculate the Top Scoring Pair (TSP) score (Δ) of all gene-pair combinations between all pair-wise class comparisons. As previously described [1], the TSP score between two classes d_m and d_n, of two genes, gene i and gene j, is defined as:

$$\Delta_{i,j}(d_m, d_n) \;=\; \left|p_{i\,>\,j}(d_m) - p_{i\,>\,j}(d_n)\right| ,$$

where $p_{i\,>\,j}(d_m)$ and $p_{i\,>\,j}(d_n)$ denotes the percentage of samples in d_m and d_n, respectively, whose expression of gene i is higher than that of gene j. $\Delta_{max}(d_m, d_n)$ denotes the maximum $\Delta_{i,j}$ between d_m and d_n over all gene pairs i and j

Let C designate an evolving set of groups of labels that starts off as the set of individual class $(d_1, \dots, d_7)$. The brain disease diagnostic hierarchy was constructed by progressively evolving C towards the set of all groupings in the hierarchy using the following steps:

1. For all pair-wise comparisons of distinct elements in C, we calculate all Δ_{max}. The leaves of the class-pair d_m and d_n with the smallest value of Δ_{max} are merged into the first node of the tree, denoted as n_{d_m,d_n}.

2. Δ_{max} of all pair-wise comparisons of the elements in the updated C are calculated, and the pair with the smallest value of Δ_{max} is grouped into the next node of the tree. Since at this point C contains one non-singleton node and a host of other leaves, the next merging can be either between two leaves d_u and d_v, denoted as n_{d_u,d_v}, or between a node $n_{d_m,d_n}$and a leaf d_u, denoted as n_{d_m,d_n,d_u}. Whichever pair with the smallest Δ_{max} merges to form a new node in C.

3. This process of finding the minimum Δ_{max} for all pair-wise elements in C, and adding the new node in C, is iterated until all nodes and leaves are connected to form a tree structure. All classes combine to form the top node $n_{d_1,\dots,d_7}$ at the top of the diagnostic hierarchy (i.e. root).

b. Identification of the node marker panel

The general idea of the node marker panel discovery method is to find a classifier at every node (excluding the root) and leaf of the *diagnostic hierarchy*. The node classifiers are based on common expression attributes of phenotypes grouped within a particular node; *these classifiers consist of a set of gene-pair binary decision rules*, whose collective 'true $(= 1)$' or 'false $(= 0)$' outcomes are to guide classification of a transcriptome test sample towards a brain phenotype. The gene-pair classifiers of the node marker panel are identified through the following steps:

1. Let X_t denote all samples included in the training of node *t*. These samples correspond to phenotypes of either $Y_t \in L_t$ or $Y_t \in L_{t^c}$, where L_t is the subgrouping of classes at *t* (we start from a child nodes of the root), and L_{t^c} represents the set of classes that do not belong to L_t. Using all expression profiles in X_t, we identify nine disjoint gene pairs (g_i, g_j) with the nine highest values of $\Delta_{i,j}$ between L_t and L_{t^c}. This set of gene pairs is denoted as $P_t = (p_1, \dots, p_9)$, where p_m is the

gene-pair with the mth highest $\Delta_{i,j}$. Here, for any given transcriptome sample, the two genes of p_m comprise the following decision rule: If a sample displays the relative expression relation $x_{g_i} > x_{g_j}$, then the sample is classified as L_t; otherwise, the sample is classified as L_{t^c}, where x_{g_i} and x_{g_j} are expression levels of gene i and gene j, respectively.

When multiple gene pairs achieve the same $\Delta_{i,j}$, the gene pairs are preferentially selected by the tie-breaking scheme employed by Tan *et al.* [1]. Note that since the classification compares L_t and L_{t^c} at each node t, the classifiers of the two child nodes of the root must necessarily be the same. This special case occurs in the child nodes of the root because every class label is represented either in the left child or the right child.

2. For the n gene pairs in P_t with the n highest TSP scores ($x = 1, \dots, 9$), a constant threshold k ($k \leq n$) is found, representing the minimum number of gene pairs required to have 'true ($= 1$)' decision rule outcomes in order to have a particular sample classified as L_t. The optimal n gene pairs and threshold k are found concurrently: n is the fewest number of gene pairs that yields classification sensitivity (percentage of X_t samples that are classified as $Y_t \in L_t$ correctly) above a desired value; k is optimized to be the threshold that results in the highest overall accuracy, i.e. percentage of all samples in X_t that are classified correctly.

3. Using the optimal n gene pairs and threshold k found in Step 2, we evaluate all n gene-pair classifiers on every sample of X_t, where each classifier's binary decision rule outcome (on each sample) is either 'true ($= 1$)' or 'false ($= 0$)'. The total number of true outcomes for each sample is denoted as μ. If $\mu \geq k$, the sample is labeled 'positive' for L_t and passes into X_{t^*} of child node t^* of t for further classifier training. However, if $\mu < k$, the sample is labeled 'negative' for L_t and does not pass into X_{t^*}. In general, most samples of $Y_t \in L_t$ passes into X_{t^*}, while most samples of $Y_t \in L_{t^c}$ do not pass into X_{t^*}. This updated collection of samples of X_{t^*} is now to be used in Step 4 by the child nodes t^*.

4. We iterate Steps 1-3 on the sibling node, on its child nodes, and so forth. The classes of L_t and L_{t^c} are dependent upon the current node. Step 2 is used to find the optimal n gene pairs and threshold k. Step 3 is used to update X_{t^*} into smaller and smaller sample subsets. We iteratively train on the samples remaining in each successive X_{t^*} until gene pairs and thresholds are found for all nodes and leaves of the diagnostic hierarchy.

c. Identification of the decision-tree marker panel

At each edge of the diagnostic hierarchy, the group of class labels of the parent node is partitioned into class labels of the two children. A classifier is chosen at each edge to direct classification to either one of the child nodes. This classifier is a gene-pair (g_i, g_j) that gives $\Delta_{max}(d_m, d_n)$ between the classes of the two child nodes, L_m and L_n. The decision rule for each classifier is: IF $x_{g_i} > x_{g_j}$, THEN classify as phenotype G_m; ELSE phenotype G_n, where x_{g_i} and x_{g_j} are expression levels of genes g_i and g_j, respectively. The collection of gene-pair classifiers at all edges of the diagnostic hierarchy is accumulated vertically into a decision-tree marker panel (**Table 2**) to guide classification toward a unique leaf in the diagnostic hierarchy. The cumulative binary outcome for an entire route, from root to leaf, delineates the disease-specific molecular signature.

d. Diagnosis of transcriptome samples

The node marker panel and the diagnostic hierarchy are used for brain phenotype classification. Specifically, the relative expression orderings of the gene-pair classifiers are used to screen for disease-specific expression patterns.

Starting at either child node of the root, we use the corresponding set of n gene pairs and constant threshold k. The value of μ for the transcriptome test sample is compared with k. If $\mu \geq k$, the sample is 'positive' for L_t and classification proceeds to both child nodes. However, if $\mu < k$, the sample is 'negative' for L_t and classification stops. We continue this process on the sibling node, on the child nodes, and so forth, unless a sample is deemed 'negative' for a particular node. A test sample can have one of three diagnostic outcomes:

1. A single disease class diagnosis, where the sample is 'positive' for all the nodes of only one entire diagnostic path.

2. No diagnosis, where the sample is 'negative' for at least one node in every path. Here, the sample is rejected from classification, and determined to be none of the brain phenotypes in the diagnostic hierarchy.

3. Multiple diagnoses, where the sample is 'positive' for all the nodes of multiple paths. In this case, the classifiers of the decision-tree marker panel are used as a tie-breaker: All gene-pair classifiers' decision rules are evaluated on the sample, and the resulting binary outcome is compared to the binary signatures of the class candidates. The brain phenotype whose binary signature matches that of the test sample is chosen as the unique diagnosis.

References

1. Tan AC, Naiman DQ, Xu L, Winslow RL, Geman D (2005) Simple decision rules for classifying human cancers from gene expression profiles. Bioinformatics 21: 3896-3904.
