# Supplementary material for: Multi-study Integration of Brain Cancer Transcriptomes Reveals Organ-Level Molecular Signatures
Source: PLoS Comput Biol. 2013 Jul 25;9(7):e1003148. doi: 10.1371/journal.pcbi.1003148 (PMC3723500; doi:10.1371/journal.pcbi.1003148)
Supplement: Text S2 — Advantages of using relative expression reversals to build classifiers. (PDF) [file pcbi.1003148.s017.pdf]

Text S2. Advantages of using relative expression reversals to build classifiers

Recently, relative expression orderings (i.e. ranks) among a small number of genes have been used for the supervised classification of disease-associated phenotypes [1-3]. The Top-Scoring Pair (TSP) classifier, which uses a single gene-pair within an expression profile that exhibits a characteristic "relative expression reversal" between two classes of interest, has been shown to be a highly accurate and robust strategy in the molecular diagnostics of a variety of human cancers [4-6]. The power of this two-gene classifier method extends beyond high classification performance; by comparing the ranked expression values of only two genes, this simple classification decision rule has remarkably straightforward biological interpretation and eliminates the need for parameter estimation and data normalization. The elimination of the need for data normalization across microarrays is advantageous in the clinic, where presumably the microarray would not be the measurement platform, because the resulting ISSAC based tests require only the genes in the classifier to be measured and do not need all the other genes to assure that a normalization is done uniformly. These are important advantages, since building complex decision rules entails the risk of over-fitting, and data preprocessing may introduce unwanted bias into the dataset. For multi-category diagnoses, we extend on the basic idea of the TSP classifier into a multi-class, coarse-to-fine search strategy [7,8].

References

1. Geman D (2004) Classifying gene expression profiles from pairwise mRNA comparisons. Statistical Applications in Genetics and Molecular Biology 3.
2. Tan AC, Naiman DQ, Xu L, Winslow RL, Geman D (2005) Simple decision rules for classifying human cancers from gene expression profiles. Bioinformatics 21: 3896-3904.
3. Lin X, Afsari B, Marchionni L, Cope L, Parmigiani G, et al. (2009) The ordering of expression among a few genes can provide simple cancer biomarkers and signal BRCA1 mutations. Bmc Bioinformatics 10.
4. Price ND, Trent J, El-Naggar AK, Cogdell D, Taylor E, et al. (2007) Highly accurate two-gene classifier for differentiating gastrointestinal stromal tumors and leiomyosarcomas. Proceedings of the National Academy of Sciences of the United States of America 104: 3414-3419.
5. Xu L, Tan AC, Naiman DQ, Geman D, Winslow RL (2005) Robust prostate cancer marker genes emerge from direct integration of inter-study microarray data. Bioinformatics 21: 3905-3911.
6. Xu L, Tan AC, Winslow RL, Geman D (2008) Merging microarray data from separate breast cancer studies provides a robust prognostic test. Bmc Bioinformatics 9.
7. Tibshirani R, Hastie T (2007) Margin trees for high-dimensional classification. Journal of Machine Learning Research 8: 637-652.
8. Fleuret F, Geman D (2001) Coarse-to-fine face detection. International Journal of Computer Vision 41: 85-107.
