# Supplementary material for: Multi-study Integration of Brain Cancer Transcriptomes Reveals Organ-Level Molecular Signatures
Source: PLoS Comput Biol. 2013 Jul 25;9(7):e1003148. doi: 10.1371/journal.pcbi.1003148 (PMC3723500; doi:10.1371/journal.pcbi.1003148)
Supplement: Text S3 — Twenty-five anatomical regions of the human brain from which normal transcriptome samples were obtained. (PDF) [file pcbi.1003148.s018.pdf]

Text S3. Twenty-five anatomical regions of the human brain from which normal transcriptome samples were obtained

Roth *et al.* [1] and Roth *et al.* (GSE7307, unpublished): Accumbens, amygdala, brain stem, caudate, cerebellum, cerebral cortex, entorhinal cortex, frontal lobe, hippocampus, hypothalamus, lateral ventricle, medulla, middle temporal gyrus, occipital lobe, optic nerve, posterior cingulate cortex, prefrontal cortex, posterior fossa, parietal lobe, putamen, superior frontal gyrus, substantia nigra, thalamus, temporal lobe, and visual cortex.

References

1. Roth RB, Hevezi P, Lee J, Willhite D, Lechner SM, et al. (2006) Gene expression analyses reveal molecular relationships among 20 regions of the human CNS. Neurogenetics 7: 67-80.
