# Supplementary material for: Multi-study Integration of Brain Cancer Transcriptomes Reveals Organ-Level Molecular Signatures
Source: PLoS Comput Biol. 2013 Jul 25;9(7):e1003148. doi: 10.1371/journal.pcbi.1003148 (PMC3723500; doi:10.1371/journal.pcbi.1003148)
Supplement: Text S4 — Global statistical enrichment analysis of gene-pair classifiers. (PDF) [file pcbi.1003148.s019.pdf]

Text S4. Global statistical enrichment analysis of gene-pair classifiers

Our discussion in the main text on selected marker-panel genes provides some insight into their role in disease. Next, rather than focusing on a limited number of genes (such as only those in our marker-panel), we extended our analysis to large sets of gene pairs that distinguish GBM from OLG. We hypothesized that utilizing more complete information would offer more direct insight into the basis of the classifiers' relative expression reversal behavior, by being able to associate their global patterns with differences between the pathophysiology underlying the two brain cancers.

For any two classes and any given set of gene pairs, the union of the genes that are expressed relatively higher (respectively, lower) in each gene pair for Class 1 will be referred to as 'gene-set *i*' (resp., 'gene-set *j*'). Thus if there are N gene pairs, then 'gene-set *i*' and 'gene-set *j*' each consist of N genes. For the top 500, 1,000, and 1,500 gene pairs (numbers arbitrarily chosen to identify major trends) that best distinguish GBM (Class 1) from OLG (Class 2), we performed an enrichment analysis on the biological process ontologies of 'gene-set *i*' (expressed relatively higher in GBM than in OLG) and on those of 'gene-set *j*' (expressed relatively lower in GBM than in OLG) to find the most consistently enriched category by *Z*-score.

Information on the biological processes and chromosome numbers of genes is available in the PANTHER database [1]. The *Z*-score is defined as:

$$Z_A = \sqrt{n}\frac{(P_A - \bar{P}_A)}{\sqrt{\bar{P}_A(1-\bar{P}_A)}}$$

where n is the total number of classifier gene pairs between two classes (e.g. 500, 1000, or 1,500) and P_A and $\bar{P}_A$ are the proportion of genes characterized by biological category (or chromosome number) A in a given gene set (e.g. gene-set i) and in the null distribution (i.e. all genes in PANTHER), respectively.

Among 18 major biological processes in the PANTHER database, 'Immunity and Defense' was the most strongly enriched biological process in gene-set i (Figure S4a). Strong enrichment in 'Immunity and Defense' for the genes expressed relatively higher in GBM reflects the frequently observed presence of chronic inflammation in highly malignant cancers [2], such as in GBM [3]. In a tumor-associated inflammatory micro-environment, immune cells penetrate inside the tumor and secrete reactive oxygen species. This can cause further oxidative DNA damage and oncogenic mutations, including amplification of oncogenes or deletion of cell-cycle regulators, and thereby facilitate cancer progression, survival, and migration. Our results show that a relatively highly inflamed tumor environment, composed of a deep infiltration of immune cells and tumor cells exhibiting functions to embattle such oxidative conditions, is the most representative pathophysiological trait that differentiates the tumors of GBM and OLG.

'Neuronal Activities' was the most enriched biological process in gene-set j, or the group of genes that are expressed lower in GBM compared to OLG (Figure S4b). This functional category includes basic activities of the nerve or neuron behavior, such as synaptic transmission, neurotransmitter release, and action potential propagation. It has been shown that GBM cells release glutamate, an amino-acid neurotransmitter [4,5]. Elevated levels of extracellular glutamate concentrations is followed by an acute degeneration and death of neurons [4-6], a process known as excitotoxicity, and is one of the underlying causes of tumor-associated epileptic

seizures and neuro-cognitive deficiencies in glioblastoma patients [7]. Therefore, this glutamate excitotoxicity in GBM can be the cause of lower normal synaptic transmission and neural function relative to OLG, as is suggested by our enrichment results for gene-set *j*. Glutamate neurotoxicity has also been implicated in other neurodegenerative diseases, including stroke and Alzheimer's disease [8].

Applying the same enrichment analysis strategy described above for chromosome number, we looked for associations between our expression data and gene copy-number alterations frequently observed in GBM and OLG. The genes in gene-set *i* and gene-set *j* were the most enriched in Chromosome 1 (Figure S4c) and Chromosome 10 (Figure S4d), respectively. The loss of Chromosome 10 is one of the most frequent genetic aberrations in GBM [9,10], causing the expression of its genes to be heavily suppressed. This offers a possible explanation for the over-representation of Chromosome 10 genes in the gene-set that is expressed relatively less in GBM ('gene-set *j*'), and thereby higher in OLG. The deletion of the short-arm of Chromosome 1 is a hallmark feature of OLG [11,12], which is what we suspect to have caused the over-representation of Chromosome 1 genes in the set that is expressed relatively less in OLG ('gene-set *i*'), and thereby higher in GBM.

The results from our global enrichment analysis in biological processes and chromosome numbers display the relative differences between the collective properties of the two diseases. This offers a holistic view of the major trends that underlie the classifiers' relative expression reversal behavior, which could not have been detected by studying the gene pairs in our marker-panel alone. It is worth noting that we did not observe these same enrichment properties in the GBM-node or OLG-node classifiers in Table 2. This reflects a clear limit to the extent of which disease properties can be explained by using only the minimal number of classifier genes, since those gene pairs were chosen only in the interest of selecting the smallest set with the highest predictive accuracy, regardless of biological relevance.

References

1. Thomas PD, Kejariwal A, Campbell MJ, Mi H, Diemer K, et al. (2003) PANTHER: a browsable database of gene products organized by biological function, using curated protein family and subfamily classification (vol 31, pg 334, 2003). Nucleic Acids Research 31: 2024-2024.
2. Coussens LM, Werb Z (2002) Inflammation and cancer. Nature 420: 860-867.
3. Murat A, Migliavacca E, Hussain SF, Heimberger AB, Desbaillets I, et al. (2009) Modulation of Angiogenic and Inflammatory Response in Glioblastoma by Hypoxia. Plos One 4.
4. Ye ZC, Sontheimer H (1999) Glioma cells release excitotoxic concentrations of glutamate. Cancer Research 59: 4383-4391.
5. Takano T, Lin JHC, Arcuino G, Gao Q, Yang J, et al. (2001) Glutamate release promotes growth of malignant gliomas. Nature Medicine 7: 1010-1015.
6. Ye ZC, Rothstein JD, Sontheimer H (1999) Compromised glutamate transport in human glioma cells: Reduction-mislocalization of sodium-dependent glutamate transporters and enhanced activity of cystine-glutamate exchange. Journal of Neuroscience 19: 10767-10777.
7. Krex D, Klink B, Hartmann C, von Deimling A, Pietsch T, et al. (2007) Long-term survival with glioblastoma multiforme. Brain 130: 2596-2606.
8. Hynd MR, Scott HL, Dodd PR (2004) Glutamate-mediated excitotoxicity and neurodegeneration in Alzheimer's disease. Neurochemistry International 45: 583-595.

9. Fults D, Pedone C (1993) Deletion Mapping of the Long Arm of Chromosome-10 in Glioblastoma-Multiforme. Genes Chromosomes & Cancer 7: 173-177.
10. Pershouse MA, Stubblefield E, Hadi A, Killary AM, Yung WKA, et al. (1993) Analysis of the Functional-Role of Chromosome-10 Loss in Human Glioblastomas. Cancer Research 53: 5043-5050.
11. Burger PC, Minn AY, Smith JS, Borell TJ, Jedlicka AE, et al. (2001) Losses of chromosomal arms 1p and 19q in the diagnosis of oligodendroglioma. A study of paraffin-embedded sections. Modern Pathology 14: 842-853.
12. Gresner SM, Rieske P, Wozniak K, Piaskowski S, Jaskolski DJ, et al. (2006) Molecular analysis of chromosome 1, 10 and 19 abnormalities in human oligodendroglial tumors: relationship between frequency of LOH grade, age and gender. Clinical Neuropathology 25: 18-24.
