# Supplementary material for: Multi-study Integration of Brain Cancer Transcriptomes Reveals Organ-Level Molecular Signatures
Source: PLoS Comput Biol. 2013 Jul 25;9(7):e1003148. doi: 10.1371/journal.pcbi.1003148 (PMC3723500; doi:10.1371/journal.pcbi.1003148)
Supplement: Text S5 — Whether ISSAC can play a role in identifying misdiagnoses. (PDF) [file pcbi.1003148.s020.pdf]

## Text S5. Whether ISSAC can play a role in identifying misdiagnoses

To know whether a misclassified sample (by ISSAC) had actually been mislabeled by the histopathologist (certainly a distinct possibility) would be a difficult task for two reasons: 1) We are not able to go back to the "questionable" tumor sample and get a re-evaluation of the clinical phenotype as we were not involved in this step; 2) Our markers were trained on all samples labeled as a particular phenotype without a sample filtering stage in our pipeline, thereby taking into consideration all possible sources of heterogeneity (including potentially mislabeled samples). We did this on purpose to not allow ourselves to reject any variance from the published studies, wanting to avoid over-fitting and overly generous accuracy estimations. These conditions make it truly difficult to determine whether a misclassified sample is due to a mislabeling by the histopathologist, or a combination of the above.
