# Supplementary material for: Multi-study Integration of Brain Cancer Transcriptomes Reveals Organ-Level Molecular Signatures
Source: PLoS Comput Biol. 2013 Jul 25;9(7):e1003148. doi: 10.1371/journal.pcbi.1003148 (PMC3723500; doi:10.1371/journal.pcbi.1003148)
Supplement: Text S6 — Reasoning for selecting only Affymetrix microarray platforms and for not using probe-specific offsets. (PDF) [file pcbi.1003148.s021.pdf]

Text S6. Reasoning for selecting only Affymetrix microarray platforms and for not using probe-specific offsets

We chose to not include samples from various platforms and technologies (e.g. other Affymetrix models, Agilent microarrays, RNA-seq) because of their inherent differences in sample preparation steps, hybridization chemistry, probeset/primer length and sequences, data pre-processing techniques, and so forth – all of which lead to poor correlation of the same features, making them not as readily comparable. This was a choice we made at the beginning of our study, as we wanted to study aspects of reproducibility not associated with platform. We do acknowledge that using Affymetrix-based datasets (specifically U133A and U133Plus2.0 array chips) is a somewhat restricted case, and that our markers for brain cancers are indeed platform specific (which we state in the manuscript). However, these platforms are still the most widely represented in GEO for brain cancers (over 50%). In addition, we do not anticipate a significant change in the main message or impact of our manuscript if we were to apply our technique to a smaller sample collection of other platforms and technologies, although, of course, the classifier features themselves will likely change.

To the best of our knowledge, there are no confirmed/validated mapping steps (or offsets) that one would routinely apply on a subset of probesets for the cross-sample or cross-platform purposes. Furthermore, a simple comparison (such as relative expression comparison used throughout this study) is invariant to any monotone pre-processing (in particular any linear transformation) of the raw expression values. However, with an offset, the outcome of the pair comparison would no longer be invariant to even scaling. Thus one would lose robustness. One of the motivations for our method, and one of the reasons relative expression comparison has worked in the past, is this invariance.
