# Supplementary material for: Multi-study Integration of Brain Cancer Transcriptomes Reveals Organ-Level Molecular Signatures
Source: PLoS Comput Biol. 2013 Jul 25;9(7):e1003148. doi: 10.1371/journal.pcbi.1003148 (PMC3723500; doi:10.1371/journal.pcbi.1003148)
Supplement: Text S7 — Candidates of brain cancer molecular signatures. (PDF) [file pcbi.1003148.s022.pdf]

Text S7. Candidates of brain cancer molecular signatures

We would like to provide a cautionary point that more work is necessary prior to any application of our results to a clinical setting. Indeed, we acknowledge that our markers can only serve as classifier candidates at this time. While we were eager to confirm our markers, we did not have brain cancer biopsies at the beginning of our study to validate by qPCR. Nevertheless, we feel that our brain cancer marker candidates, and methods used for their discovery presented herein, are valuable resources to those interested in identifying novel molecular signatures from high-throughput biomolecular data.
